# The Search for Therapeutic Bacteriophages Uncovers One New Subfamily and Two New Genera of *Pseudomonas*-Infecting *Myoviridae*

**DOI:** 10.1371/journal.pone.0117163

**Published:** 2015-01-28

**Authors:** Marine Henry, Louis-Marie Bobay, Anne Chevallereau, Emilie Saussereau, Pieter-Jan Ceyssens, Laurent Debarbieux

**Affiliations:** 1 Institut Pasteur, Molecular Biology of the Gene in Extremophiles Unit, Department of Microbiology, Paris, France; 2 Institut Pasteur, Microbial Evolutionary Genomics Unit, Department of Genomes and Genetics, Paris, France; 3 CNRS, UMR3525, Paris, France; 4 Université Pierre et Marie Curie, Cellule Pasteur UPMC, Paris, France; 5 Université Paris Diderot, Sorbonne Paris Cité, Cellule Pasteur, Paris, France; 6 Laboratory of Gene Technology, Division of Gene Technology, Katholieke Universiteit Leuven, Heverlee, B-3001, Belgium; 7 Unit of Bacterial Diseases, Scientific Institute of Public Health (WIV-ISP), Brussels, Belgium; Institute of Immunology and Experimental Therapy, Polish Academy of Sciences, POLAND

## Abstract

In a previous study, six virulent bacteriophages PAK_P1, PAK_P2, PAK_P3, PAK_P4, PAK_P5 and CHA_P1 were evaluated for their *in vivo* efficacy in treating *Pseudomonas aeruginosa* infections using a mouse model of lung infection. Here, we show that their genomes are closely related to five other *Pseudomonas* phages and allow a subdivision into two clades, PAK_P1-like and KPP10-like viruses, based on differences in genome size, %GC and genomic contents, as well as number of tRNAs. These two clades are well delineated, with a mean of 86% and 92% of proteins considered homologous within individual clades, and 25% proteins considered homologous between the two clades. By ESI-MS/MS analysis we determined that their virions are composed of at least 25 different proteins and electron microscopy revealed a morphology identical to the hallmark *Salmonella* phage Felix O1. A search for additional bacteriophage homologs, using profiles of protein families defined from the analysis of the 11 genomes, identified 10 additional candidates infecting hosts from different species. By carrying out a phylogenetic analysis using these 21 genomes we were able to define a new subfamily of viruses, the *Felixounavirinae* within the *Myoviridae* family. The new *Felixounavirinae* subfamily includes three genera: *Felixounalikevirus, PAK_P1likevirus* and *KPP10likevirus*. Sequencing genomes of bacteriophages with therapeutic potential increases the quantity of genomic data on closely related bacteriophages, leading to establishment of new taxonomic clades and the development of strategies for analyzing viral genomes as presented in this article.

## Introduction

In its first report on antibiotic resistance (published on March 31, 2014) the World Health Organization pointed out that, everyone on the planet is now at risk of infection by untreatable multidrug resistant (MDR) bacterial infections (http://www.who.int/iris/bitstream/10665/112642/1/9789241564748_eng.pdf). Proposed solutions to this worldwide threat to public health include better hygiene, access to clean water, infection control in health-care facilities, vaccination and control of antibiotic prescriptions. In addition to these available solutions, discovery of new antibacterial drugs is strongly encouraged. Among current and future solutions, phage therapy—the use of bacteriophages (viruses infecting bacteria) to treat bacterial infections—occupies a singular place. This therapeutic treatment started to be used to treat human bacterial infections before the discovery of antibiotics but was later discontinued in most countries except in Eastern Europe where it was and is still regularly used, in particular in Georgia, Poland and Russia [1,2]. Facing the need for new antibacterial weapons, interest in phage therapy has reignited in the past few years, with an increasing number of publications reporting on the curative efficacy of bacteriophages in various animal models of infection. However, these reports are not always accompanied by molecular studies of the therapeutic bacteriophages.

Nowadays, the reduced cost of sequencing is an incentive to provide access to the raw molecular data of therapeutic bacteriophages [3]. However, analysis of these data is still a major challenge due to the poor conservation of sequences between bacteriophage genomes. In addition, each bacteriophage contains several “orphans” to which a function is difficult to assign. In most cases, a rapid analysis of bacteriophage genomes can determine whether a bacteriophage is temperate or virulent. This is valuable information because temperate bacteriophages would not be recommended for therapeutic use due to their capabilities to exchange genetic material with bacterial strains [4]. Nevertheless, beyond such analysis, one can distinguish between two situations: either a genome reveals its close proximity to published genomes, or no close homolog can be found making it rather difficult to assign a classification with confidence and to elaborate a strategy for molecular characterization. We hypothesize that comparative genomics of closely related bacteriophages may both help their accurate classification and highlight molecular characteristics which could be used to guide further analysis.

From our previous work, we isolated in 2006 and 2009 bacteriophages infecting *Pseudomonas aeruginosa*, a Gram-negative opportunistic pathogen widespread in the environment [5–7]. *P. aeruginosa* is acknowledged as the leading cause of chronic infections in cystic fibrosis patients. It is frequently isolated in cases of ventilation-associated pneumonia, chronic obstructive pulmonary disease, and also on the skin of burns patients and other sites such as urinary tract and ears [8–10]. The therapeutic potential of these bacteriophages was then evaluated using a mouse model of acute lung infection and some were also included in a preclinical study performed on cystic fibrosis sputa samples [5,6,11,12]. The genomes of two of these bacteriophages, namely PAK_P1 and PAK_P3, were sequenced in 2009 and published in 2010 and 2011 respectively, revealing no close relationship to any other published bacteriophage genomes [5,6]. The mass spectrometry of major capsid proteins of these bacteriophages led us to identify a distant homology (less than 30% identity) to the major capsid protein of Felix O1 bacteriophage [5,6]. The relationship to Felix O1 bacteriophage has never been characterized further despite the publication of four other closely related *P. aeruginosa* bacteriophages, namely KPP10, JG004, PaP1 and vB_PaeM_C2-10_Ab1 [13–16]. We report here on the genome sequences of four additional bacteriophages isolated by our group (namely PAK_P2, PAK_P4, PAK_P5 and CHA_P1) and describe their genome organization through the comparison with other closely related bacteriophages infecting *P. aeruginosa*. Our findings, based on analysis of protein family profiles led us to develop a coherent bacteriophage taxonomy comprising two new genera, and new subfamily of viruses (tentatively named *Felixounavirinae*) within the *Myoviridae* family. Identification of some original and intriguing molecular characteristics was also successful.

## Results

### Isolation of six new bacteriophages infecting *P. aeruginosa*

**Figure 1:**
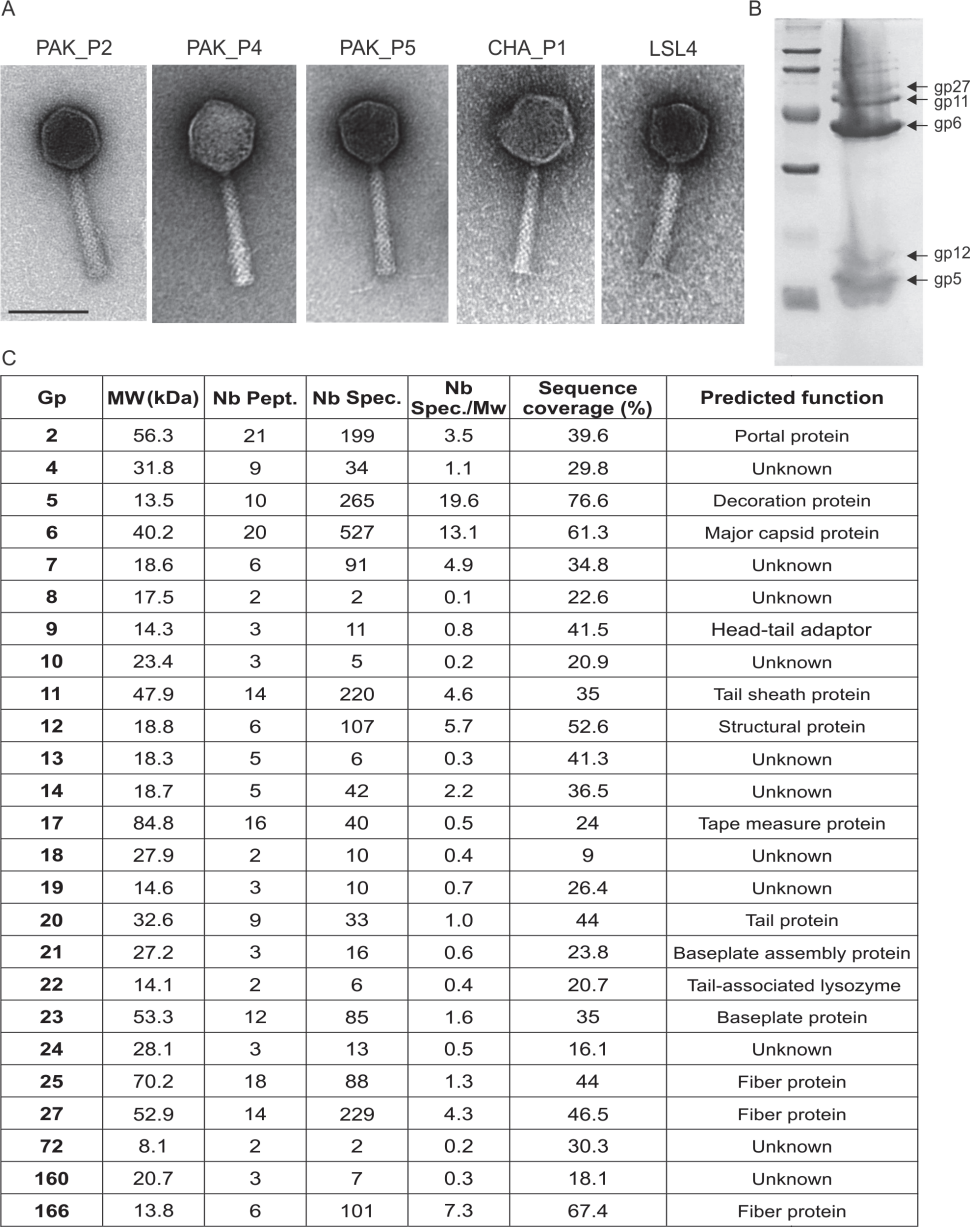
Electron microscopy of bacteriophages and proteomic analysis of PAK_P3 virion. A. Electron micrographs of the indicated bacteriophages (scale bar: 100 nm). B. Denaturing polyacrylamide gel of PAK_P3 virion proteins. C. Proteins identified by mass spectrometry analysis (ESI-MS/MS). MW, theoretical molecular weight; Nb Pept., number of unique peptides identified; Nb Spec., total number of spectra. Nb Spec/Mw, relative abundance; Sequence coverage, percentage of the protein sequence covered by peptides

In 2006 we isolated five bacteriophages infecting the PAK strain of *P. aeruginosa* (PAK_P1, PAK_P2, PAK_P3, PAK_P4 and PAK_P5) and, in 2009, a bacteriophage (CHA_P1) infecting the CHA strain, a cystic fibrosis isolate [17]. Electron microscopy showed that these six bacteriophages had a similar morphology (see Fig. 1A for images of previously unpublished bacteriophages), with an icosahedral head and criss-cross pattern on the tail, characteristics resembling FelixO1 bacteriophage infecting *Salmonella* [18]. Despite most of them having been isolated on the same host, a restriction fragment length polymorphism analysis revealed that their genomic content was not identical (not shown). The genomes of these six bacteriophages were then sequenced and a Megablast analysis revealed their close similarity to five other bacteriophages infecting also *P. aeruginosa*, JG004 [13], PaP1 [15] vB_PaeM_C2-10_Ab1 [14], KPP10 [16] and LSL4 [19]. An additional bacteriophage, P3_CHA [6], was excluded from this study because the difference between its DNA sequence and that of PAK_P3 was negligible (2 nucleotides). Detailed annotation of the genomes of the six newly sequenced bacteriophages is provided as supplementary information (S1 Text). Briefly, we found that 10 to 15% of the predicted ORFs could be linked to a putative function while 5 to 10% displayed no similarity to any other sequence in current databases. About 50% of each bacteriophage genome displays sequence similarity only to genes encoding hypothetical proteins (unknown functions) present only in the 11 bacteriophages listed above. The remaining ORFs were also annotated as encoding hypothetical proteins but presented similarities to ORFs from mostly bacteriophage or prophage genomes. Position of genome termini of the six bacteriophage genome sequenced was identified using sequence coverage (see S1 Table and S1 Text) [20,21].

### Genomic and proteomic analysis of PAK_P1-like and KPP10-like bacteriophages

Several characteristics (genome length, GC content and number of tRNA) of the 11 bacteriophages mentioned above suggest they could be classified into two distinct clades (Table 1). We decided to name these proposed two clades according to the publication date of the genome of the first bacteriophage discovered in each clade: “PAK_P1-like” (including PAK_P1 published in 2010, PAK_P2, PAK_P4, JG004, PaP1 and vB_PaeM_C2-10_Ab1) and “KPP10-like” (including KPP10 published in 2011, PAK_P3, PAK_P5, CHA_P1 and LSL4).

**Table 1:** General characteristics of the genomes of the 11 bacteriophages belonging to either the PAK_P1-like or the KPP10-like clades.

Clade	Name	Length	ORFs predicted	GC content	t-RNA	Accession	Reference
PAK_P1-like	PAK_P1	93398	181	49.50%	13	KC862297	[5]
	PAK_P2	92495	176	49.30%	11	KC862298	[11]
	PAK_P4	93147	174	49.30%	13 + 1 pseudo	KC862300	[11]
	JG004	93017	161	49.30%	12	NC_019450.1	[13]
	C2-10_Ab1	92777	158	49.28%	12	NC_019918.1	[14]
	PaP1	91715	157	49.40%	13	NC_019913.1	[15]
KPP10-like	KPP10	88322	146	54.80%	3	NC_015272.1	[16]
	PAK_P3	88097	165	54.80%	3	KC862299	[6]
	PAK_P5	88789	164	54.70%	3	KC862301	[11]
	CHA_P1	88255	166	54.60%	3	KC862295	[11]
	LSL4^a^	87739	165	54.80%	3	Not published	[19]

a: the LSL4 bacteriophage was isolated in Sri Lanka, with the Lio12 strain, and its genome sequence was kindly provided by R. Lavigne.

**Figure 2:**
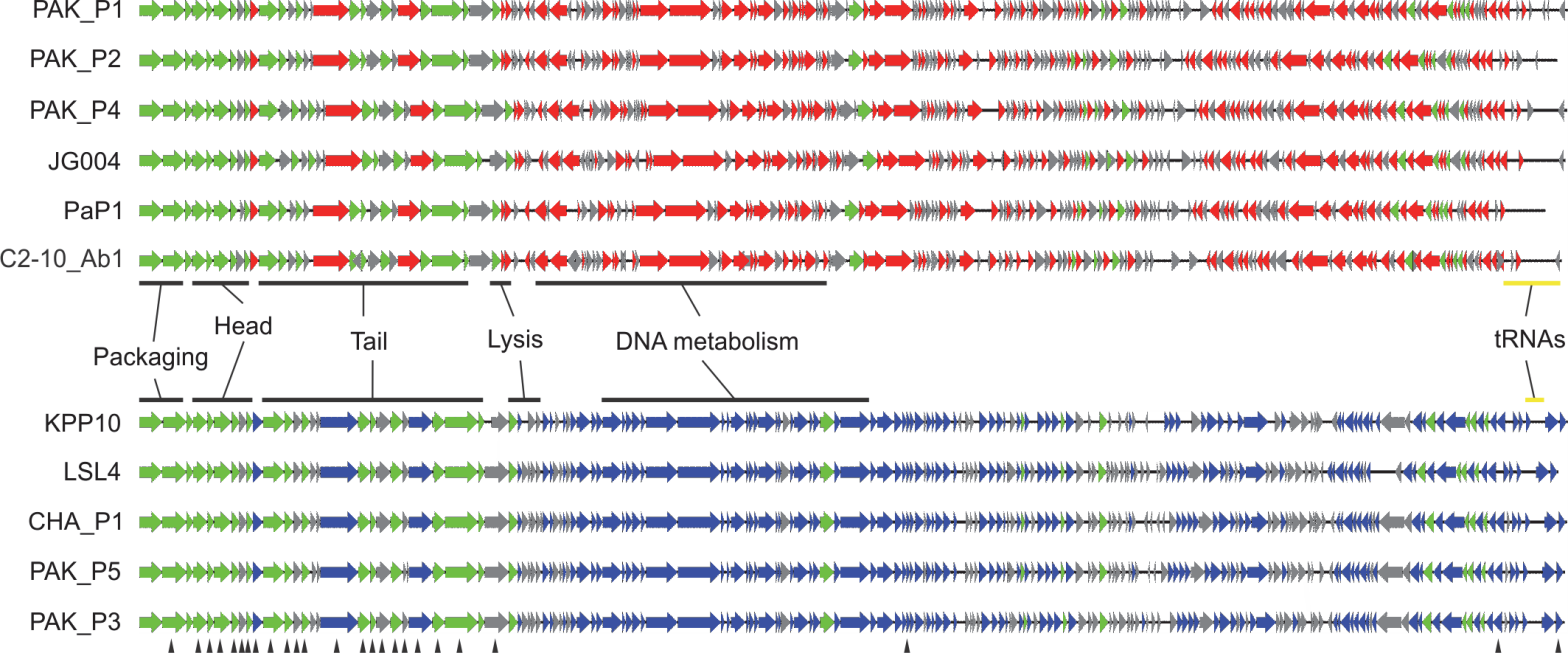
Maps of the 11 genomes involved in this study. In green, the core ORFs homologous in the 11 bacteriophages; red and blue ORFs are common to the PAK_P1-like and KPP10-like bacteriophages, respectively. Arrows (▲) designate the sequences corresponding to proteins identified by mass spectrometry of the PAK_P3 virion.

PAK_P1-like bacteriophages have a mean genome size of 92.8 kb (SD = 598 bp), a mean GC% of 49.3% (SD = 0.09%) and carry 11 to 13 tRNAs. By contrast, KPP10-like bacteriophages have a mean genome size of 88,2 kb (SD = 345 bp), a mean GC content of 54.8% (SD = 0.08%) and only three predicted tRNAs: a tRNA-Asn, a tRNA-Tyr and a tRNA-Gln. In both clades GC content is significantly lower than that of the *P. aeruginosa* host (67% GC for the *P*. *aeruginosa* core genome [22–24]), consistent with previous observations that bacteriophages tend to have a higher proportion of A+T residues than their bacterial host [25]. Most bacteriophages contain one or two tRNAs, but a few (including bacteriophage FelixO1) have been shown to contain more than 20 [26]. In both clades, the tRNAs were found to be located in the close vicinity of the packaging ORFs, upstream from the large terminase subunit, spanning regions of 2.52 kb (PAK_P1-like) and 400 bp (KPP10-like) (Fig. 2). The codon usage of the representative bacteriophages PAK_P1 and PAK_P3 was compared with that of their isolation host (the PAK strain; S2 Table). We found that, in PAK_P1, 13 tRNAs correspond to codons used more frequently than in the host, with tRNA-Leu and tRNA-Arg being the most frequent (respectively 16 and 29 times more). By contrast, in PAK_P3, a higher frequency was found only for the tRNA-Gln.

Published results of a proteomic analysis of PaP1 bacteriophage (member of the PAK_P1-like clade) reported the identification of 12 proteins while a preliminary report on KPP10 (member of the KPP10-like clade) reported only 7 proteins. In order to obtain additional identification of structural proteins from a bacteriophage of the latter clade we analyzed the virion proteins from PAK_P3 (Fig. 1B). A total of 25 proteins were identified using ESI MS/MS analysis (Fig. 1C). Twenty one of them are encoded within the structural region of the genome, two (gp160 and gp166) were relatively close to it and one, gp72, was not. As the abundance of Gp72 was low, this protein may display some affinity for structural proteins, rather than directly taking part in the virion assembly. Gp6, the major capsid protein, was the most abundantly identified protein. Notably, the head decoration protein Gp5 had the highest relative abundance (#spectra/mol. weight) suggesting a prominent role in PAK_P3 capsid morphology.

### Identification of putative regulatory elements

**Figure 3:**
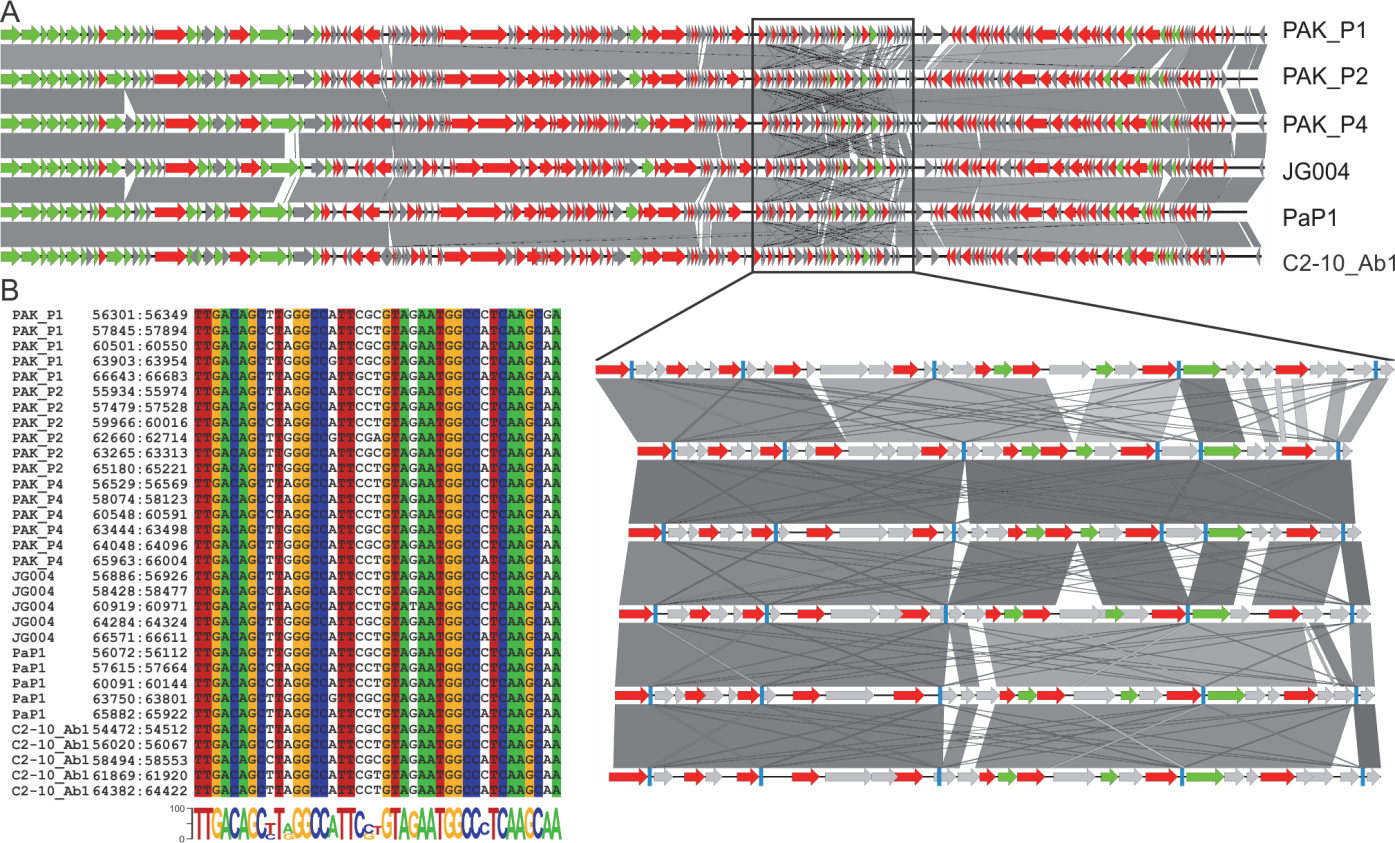
Identification of repeated promoter-like regions of the PAK_P1-like clade. A. Representation of the homology (blastn) between the six PAK_P1-like bacteriophages, with an enlargement of the region in which 41-nt repeats were identified (represented as light blue boxes). B. Alignment of the total of 32 repeats identified in the six genomes, with colors indicating strictly conserved bases, and a WebLogo representation of the consensus (the height of the letters represents their frequency at each position) below the alignment.

Alignments of the nucleotide sequences of bacteriophage genomes of PAK_P1-like clade revealed the presence of a variable region which is 11 kb long (approximately 12% the length of the genome) (Fig. 3A). A blast analysis and the subsequent alignment of regions from all the PAK_P1-like bacteriophages revealed the presence of a 41 nucleotide-long repeated sequence (5 repeats in PAK_P1, PaP1, vB_PaeM_C2-10_Ab1, JG004 and 6 repeats in PAK_P2, PAK_P4). Alignment of the entire set of repeats from the PAK_P1-like clade revealed that 32 of the 41 nucleotides were strictly conserved (Fig. 3B). As this intergenic motif contains a conserved σ^70^ promoter sequence (TTGACA-N_17_-TAgAAT), it most likely serves to guide the bacterial RNA polymerase to the early phage genes at the onset of phage infection. Similar promoter repeats were identified in the putative early genome regions of the KPP10-like clade, but at a much lower frequency, with each member of this clade containing only two repeats (S1A Fig.). More unusual is the perfect nucleotide conservation surrounding the-35 and-10 motifs. In a blastn search against the nonredundant database, the consensus sequence of these repeats yielded hits only with bacteriophage genomes from these two clades, with an e-value < 0.0001 (the consensus sequence for repeats of the KPP10-like clade is more conserved; S1B Fig.). Therefore, these repeats are an additional characteristic to these two clades which could be linked to the common bacterial species they infect.

### Designation of core genes and identification of specific markers

We determined the proportion of gene products conserved in the PAK_P1-like and KPP10-like clades, by carrying out a comparative genomics analysis with a 40% similarity threshold and a size constraint (Materials and Methods). The two clades were well delimited, with a mean of 86% and 92% of proteins considered homologous within individual clades, and 25% proteins considered homologous between the two clades (S3 Table).

We used these parameters to define protein families, for the subsequent creation of protein profiles for screening against all publicly available bacteriophage sequences, to perform our annotation (Materials and Methods). In total, 404 protein families (S4 Table) were identified, several of which included ORFs specific to only the PAK-P1-like or KPP10-like clade of bacteriophages (represented in red and blue, respectively, in Fig. 2). Many of these ORFs appeared to be conserved between the two clades but were nevertheless too divergent to include them in the set of 26 “core families” with a homolog in each of the 11 bacteriophages. The ORFs corresponding to these 26 families were designated as the core genes of the 11 bacteriophages (S5 Table). Unsurprisingly, most of these core genes belong to the structural module of these genomes (Fig. 2). Despite the search using protein profiles very few predicted ORFs could be assigned with confidence to a function. We therefore carried out additional analyses (transmembrane domains and structural similarities), including iterative searches with the alignment of the 11 homologs from each family (S1 Text). Nevertheless, the majority of ORFs still could not be assigned to a function highlighting the novelty of the bacteriophages.

We then searched the 11 bacteriophage genomes for markers that could be used to detect in a specific manner bacteriophages infecting *P*. *aeruginosa* belonging to the two clades. Four core families were then selected (families 5, 21, 22 and 25 in S5 Table). The corresponding ORFs from the 11 genomes showed no significant matches (e-value > 0.001) with other elements in blast searches against the nonredundant database. Alignments of ORFs from family 5 displayed regions of strong identity, which we used to design specific degenerated primers that were then tested experimentally (5′–CATCAGCGYCTKAGCAACTGGCT–3′ and 5′–CTGGTSWACYGCGAAGATGTTCT–3′). The detection of as few as 100 pfu of PAK_P1 was achieved in solution containing 1x10^7^ pfu of PhiKZ, an unrelated *Myoviridae* phage infecting *P*. *aeruginosa* (no PCR product was obtained when using a solution containing only PhiKZ). This set of primers should therefore be sufficient to detect the presence of a bacteriophage from these two clades (we also obtained a PCR product for PAK_P5). Sequencing of the PCR products allows further assignment of the bacteriophage to one of the two clades.

### Evolutionary relationships of bacteriophage genomes related to the *Felixounalikevirus* genus

We then attempted to characterize relationships between the bacteriophages of both the PAK_P1-like and KPP10-like clades and more distantly related bacteriophages. Using HMMER we built sequence profiles for the families of homologous proteins defined in the 11 bacteriophages and searched for homologous proteins among the bacteriophage genomes of GenBank. We identified 10 bacteriophages containing a number of genes (>20) with significant matches (e-value<0.001). These 10 additional bacteriophages belong to *Myoviridae* family of viruses and have genomes larger than 84 kb (Table 2).

**Table 2:** General characteristics of the 10 bacteriophages most closely related to *Pseudomonas*-infecting bacteriophages of PAK_P1-like and KPP10-like clades.

Clade	Name	Host	Size (bp)	Homologous proteins^a^ (%)	Genome ID
FelixO1-like	phiEa104	Erwinia	84565	28 (24%)	NC_015292
	phiEa21-4	Erwinia	84576	26 (22%)	NC_011811
	WV8	Escherichia	88487	27 (19%)	NC_012749
	FelixO1	Salmonella	86155	28 (21%)	NC_005282
rV5-like	PVP-SE1	Salmonella	145964	38 (16%)	NC_016071
	vB_CsaM_GAP31	Cronobacter	147940	37 (14%)	NC_019400
	vB_EcoM-FV3	Escherichia	136947	28 (13%)	NC_019517
	CR3	Cronobacter	149273	30 (11%)	NC_017974
	rV5	Escherichia	137947	27 (12%)	NC_011041
	ICP1	Vibrio	125956	21 (9%)	NC_015157

^a^ indicates the number of homologous proteins identified with HMMER (e-value<0.001) by comparison to the total set of protein families of the 11 PAK_P1-like and KPP10-like bacteriophages.

(%) indicates the number of homologous proteins identified divided by the total number of proteins encoded by each genome.

**Figure 4:**
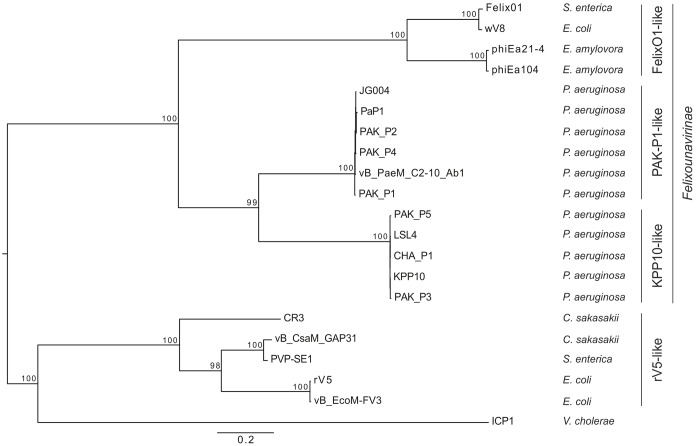
Phylogenetic tree of PAK_P1-like and KPP10-like bacteriophages and their closest relatives. The maximum likelihood tree was built from a concatenated alignment of three core proteins (predicted to encode the portal protein, the major capsid protein and the tail sheath protein) common to the 21 bacteriophages. Bootstrap values are indicated and the tree was rooted on the midpoint root.

In the set of 21 genomes we identified three conserved genes predicted to encode structural proteins (portal, major capsid and tail sheath). The corresponding protein sequences were then aligned, concatenated and a phylogenetic tree was built using the maximum likelihood method (see Materials and Methods). Not surprisingly, the two PAK_P1-like and KPP10-like clades of bacteriophages were found to be most closely related to each other (Fig. 4). The closest clade to those is constituted by the four bacteriophages belonging to the *Felixounalikevirus* genus (FelixO1, wV8, phiEa21-4 and phiEa104). These four bacteriophages have several features in common with the PAK_P1-like and KPP10-like bacteriophages, including a large number of tRNAs (>20), similar genome sizes (~90 kb) and almost identical morphologies. These findings strongly suggest that these three clades are related. An analysis with the CoreGenes program, which is used to define bacteriophage taxonomic groups [18,27], indicated that FelixO1 had only 22 and 21 homologous proteins in common with PAK_P1 and PAK_P3 respectively. These values, corresponding to 14 and 11% of the respective proteomes of these bacteriophages are well below the 40% shared proteins used to define a genus, which confirms that these clades belong to different genera [18]. The second closest clade to the PAK_P1-like and KPP10-like clades contained five bacteriophages, infecting various *Enterobacteria*, which belong to a proposed rV5-like viruses genus (CR3, vB_CsaM_GAP31, PVP-SE1, rV5 and vB_EcoM-FV3) [28,29]. Finally, the most distantly related bacteriophage was the *Vibrio* bacteriophage ICP1. This molecular phylogeny analysis therefore revealed that the *Cronobacter* bacteriophages vB_CsaM_GAP31 and CR3, the *Salmonella* bacteriophage PVP-SE1 and the *Escherichia coli* bacteriophages FV3 and rV5 are closely related to each other, forming an rV5-like genus divergent from *Felixounalikevirus*, PAK_P1-like and KPP10-like bacteriophages and from the *Vibrio* bacteriophage ICP1 (Fig. 4) [28]. It has recently been suggested that this rV5-like genus could be split into three separate genera, rV5-like viruses (rV5 and FV3), PVP-like viruses (PVP-SE1, GAP31 and SSE-121, an as yet unpublished genome from A. Letarov) and Phi92-like viruses [30], which is consistent with our molecular phylogeny analyses.

Horizontal exchange may however affect the organization of bacteriophage genomes and blur phylogenetic reconstructions. As a consequence, genes located in different functional modules may have different evolutionary histories [31]. We therefore used a conserved nonstructural protein, the primase, to reconstruct the phylogeny of 19 of these bacteriophages (no homolog of the primase was identified in PVP-SE1 and vB_CsaM_GAP31). The phylogenetic tree obtained with primase sequences confirmed the phylogeny based on structural proteins (S2 Fig.). These results suggest that PAK_P1-like and KPP10-like bacteriophages are related to bacteriophages infecting different hosts. These results do not support relationships of these two clades with other *Pseudomonas*-infecting bacteriophages as proposed by Lu *et al*. [15].

## Discussion

Worldwide reports on MDR infections and the lack of new antibacterial drugs led to the reevaluation of phage therapy. While *in vivo* data from various animal models are encouraging, molecular studies on candidate therapeutic bacteriophages are still scarce. Following a recent report on *in vitro* and *in vivo* evaluations of six bacteriophages infecting *P*. *aeruginosa* (PAK_P1, PAK_P2, PAK_P3, PAK_P4 and PAK_P5 and CHA_P1), we performed the *in silico* analysis of these bacteriophage genomes. They were found to be closely related to another five genomes present in the database which infect *P*. *aeruginosa* (JG004, PaP1, vB_PaeM_C2-10_Ab1, KPP10 and LSL4). Comparative analysis of the general characteristics of these 11 genomes (GC content, genome length, number of tRNAs) suggested that they constitute two clades named PAK_P1-like and KPP10-like viruses. Consistent with these characteristics, the bacteriophages of these two clades share less than 40% of proteins, despite displaying an almost identical genome organization [18]. It is worth noting that, during the revision of this manuscript, the genome of PhiPsa374, a bacteriophage infecting *Pseudomonas syringae*, was published as being closely related to PAK_P1 and JG004 bacteriophages [32], which suggests it would belong to the PAK_P1 like clade.

An additional characteristic of these 11 bacteriophages was identified with the repetition of promoter-like sequences located in a short region which could correspond to a putative early transcribed region. The consensus sequences of these repeats are very specific suggesting that these bacteriophages may use a particular way to regulate gene expression. As no homologs to these sequences were found in PhiPsa374 bacteriophage infecting *P*. *syringae*, it is tempting to speculate that they may be specific to *P*. *aeruginosa*.

The 11 bacteriophages were isolated in various countries (France, Japan, Germany, China and Ivory Coast), at different time periods, from different hosts, but their genomes did not display mosaic structure frequently observed in genomes of bacteriophages [31,33]. This may be due to the lack of recombinases, which are involved in genome mosaicism [34–37]. Finally, in blast searches against the nonredundant database (last check in June 2014) with primer sequences designed to detect specifically bacteriophages belonging to PAK_P1-like or KPP10-like clades, no matches outside this group of 11 bacteriophages were obtained. This suggests that either these primers are too stringent (they indeed excluded PhiPsa374) or that bacteriophages belonging to these clades are not abundant and not yet represented in virome data.

Genomic analysis of the 11 bacteriophages led to the definition of protein profiles, which are more sensitive than sequence-sequence comparisons for the detection of distantly related homologs [38]. This is particularly important when viruses infect different species, as the tendency towards sequence adaptation to hosts leads to considerable divergence. Indeed, we identified 10 distantly related bacteriophages infecting various hosts (Felix O1, wV8, phiEa21-4, phiEa104, CR3, vB_CsaM_GAP31, PVP-SE1, rV5, vB_EcoM-FV3 and ICP1). We were then able to place the two new clades in the virus classification, using three conserved structural proteins to reconstruct phylogeny, revealing new relationships between the entire set of 21 bacteriophages. This reconstruction of phylogeny, in addition to the proteome comparisons of PAK_P1 and PAK_P3 with Felix O1, provided clear support for the creation of two new genera: *PAK_P1likevirus* (including PAK_P1, PAK_P2, PAK_P4, JG004, PaP1 and vB_PaeM_C2-10_Ab1) and *KPP10likevirus* (including KPP10, PAK_P3, PAK_P5 and CHA_P1). We suggest that these two genera could, together with the *FelixO1likevirus* genus (including Felix O1, wV8, phiEa21-4 and phiEa104), be grouped into a new subfamily of the *Myoviridae* named *Felixounavirinae*. Our results also showed that classification methods based on gene content [39,40], give a reliable information despite the limitations imposed by the rapid evolution of divergent sequences in bacteriophage genomes [41,42].

Our *in silico* analysis not only revealed new relationships between bacteriophages but also paves the way for a better molecular characterization of these viruses which display a clear therapeutic potential. Our results clearly highlighted the most conserved genetic elements which could represent the first targets for in depth molecular characterization. For example, out of the nine core genes not associated with putative functions, three (family number 21, 22 and 23) are located outside the structural region and therefore are most likely to carry essential functions for these viruses. Additional molecular analysis based on our results should provide insights on whether both genera rely on similar molecular processes to hijack their host, on the molecular basis of the differences in efficacy observed *in vitro* and *in vivo* towards a same host, or on the specific genes needed for infecting other hosts than *P*. *aeruginosa* (both closely related like *P*. *syringae*, or more distant like *Salmonella*, *E*. *coli* and *Erwinia)*. In addition, it would be interesting to check whether the conclusions drawn from other comparative genomics of *P*. *aeruginosa* bacteriophages belonging to a different genera, such as the one conducted on PhiKMV bacteriophages on host range and antibodies neutralization, apply to the two new genera [43].

To conclude, it seems likely that years will be needed to achieve a complete molecular characterization of new bacteriophages, since such work has not even yet been completed for model bacteriophages such as T4 or T7. From a clinical perspective, with an increasing number of patients running out of antibiotics-based solutions, the question of the extent to which novel bacteriophages, positively evaluated for their therapeutic potential, should be further characterized before being used in medicine is raised [4,44–46].

## Materials and Methods

### Sequencing and annotation of bacteriophage genomes

Accession numbers of genomes used in this study are reported in Table 1.

The genomes of the PAK_P1, PAK_P2, PAK_P3, PAK_P4, PAK_P5 and CHA_P1 bacteriophages were sequenced by the 454 technique. The various contigs were assembled with Sequencher software (v4.8, Gene Codes Corporation, Ann Arbor, MI, USA). The genome sequences obtained were then submitted to the Phage RAST program [47] and manually curated in the Artemis Genome Browser [48], with NCBI blast tools (blastp, blastn, tblastx and psi-blast). We identified tRNAs with the tRNA-Scan SE online tool, using the default search mode and the same settings as for a bacterial source [49].

### Bioinformatic analysis

For clarity, we present all genome alignments with an arbitrary start at the first base of the ORF predicted to encode the large terminase subunit, but we retained the gene identifiers for genomes already published. MegaBlast analysis was used to identify related bacteriophage genomes. Transmembrane domains were predicted with TMHMM Prediction Server (www.cbs.dtu.dk/services/TMHMM). Structural similarity searches were performed with HHPred [50]. Genome maps were generated with Easyfig [51]. Sequence coverage was assessed with Tablet software [52].

### Comparative genomics analysis

Homologous proteins were defined as proteins displaying >40% similarity and a difference of <50% in protein length. A similarity score was calculated with the BLOSUM60 matrix and the Needleman-Wunsch end gap-free alignment algorithm (in house software). Families of homologous proteins were then built by transitivity: a protein belongs to the family if homologous to a protein already present in this family. Sequences were aligned with MUSCLE v3.6 [53] and protein profiles were built with HMMER [54] for each protein family. We then used HMMER to compare each protein family profile with a set of complete genome sequences for 831 nonredundant bacteriophages, downloaded from GenBank (ftp://ftp.ncbi.nih.gov/genomes/Viruses/ last accessed April 2013). Bacteriophages presenting at least 20 positive matches (e-value<0.001) were retained for phylogenetic analysis.

### Phylogenetic analysis

We assessed the relationship between the 21 bacteriophages, defining homologous proteins as described above, but with a lower threshold (>35% similarity and <50% of difference in protein length). Three homologous proteins were found to be common to all 21 bacteriophage genomes and were used to infer their relationships. We first aligned the sequences for each group of homologous proteins independently, with MUSCLE v3.6 [53]. Non-informative positions were trimmed with BMGE, using the BLOSUM30 matrix [55]. The three alignments were then concatenated into a single alignment and a maximum likelihood tree was built with PhyML v3.0 with a LG + Γ(4) model [56]. The topology of the tree was determined with 100 bootstrap replicates, under the same model. The tree was rooted on the midpoint root.

### Electron microscopy and proteomic analysis

Cesium chloride-purified bacteriophage preparations were used for electron microscopy studies in a JEOL 1200 EXII electron microscope, after staining with uranyl acetate. Proteomic analysis of the PAK_P3 virion was performed as previously described [57,58]. Briefly, heat-denaturated virions proteins were separated onto a SDS-PAGE gel which was stained with Coomassie. Bands were excised and stain removed before reduction by DTT (101mM), followed by alkylation with iodoacetamide (55mM). Following trypsin digestion peptides were analyzed by electrospray ionisation tandem mass spectrometry (ESI MS/MS).

## Supporting Information

S1 Text This text contains details about annotation and genome termini of PAK_P1, PAK_P2, PAK_P3, PAK_P4, PAK_P5 and CHA_P1 bacteriophages. (DOCX)

S1 Table Sequences and coordinates of the boundaries of regions covered by high numbers of reads of the six bacteriophage genomes reported in this study. (PDF)

S2 Table Comparison of the codon usage of bacteriophages PAK_P1 and PAK_P3 with that of their host (strain PAK) (PDF)

S3 Table Percentage of ORFs homologous between bacteriophages of the PAK_P1-like and KPP10-like clades (PDF)

S4 Table List of the 404 protein families deduced from the genomes of PAK_P1-like and KPP10-like bacteriophages (PDF)

S5 Table Families of core ORFs and families with putative identified functions of PAK_P1-like and KPP10-like bacteriophages. Question marks indicate that only one analysis provided support for the indicated putative function, while absence of question marks indicate that at least two analysis were concordant.
(PDF)

S1 Fig Identification of repeated promoter-like regions of the KPP10-like bacteriophages A. Representation of the homology (blastn) between the five KPP10-like bacteriophages in which 41-nt repeats were detected (represented as light blue boxes). B. Alignment of the 10 repeats identified in the five genomes, with colors indicating strictly conserved bases, with a WebLogo representation of the consensus (the height of the letters represents their frequency at each position) shown beneath the alignment.
(PDF)

S2 Fig Phylogenetic tree for the primase of PAK_P1-like and KPP10-like bacteriophages and their closest relatives. The maximum likelihood tree was built from a protein alignment of the primase sequences, a non-structural protein, common to 19 bacteriophages (the primase was not identified in the PVP-SE1 and GAP31 genomes). Bootstrap support is indicated on the tree. The tree was rooted on the midpoint root.
(PDF)
