# Supplementary material for: The Search for Therapeutic Bacteriophages Uncovers One New Subfamily and Two New Genera of Pseudomonas-Infecting Myoviridae
Source: PLoS One. 2015 Jan 28;10(1):e0117163. doi: 10.1371/journal.pone.0117163 (PMC4309531; doi:10.1371/journal.pone.0117163)
Supplement: S1 Text — (DOCX) [file pone.0117163.s001.docx]

**Supplementary data**

**Annotation of nucleotide- and DNA metabolism-associated functions**

The ORFs constituting the family 28 (Table S5) encoded proteins that are similar to proteins predicted to be RNA polymerases in *Pseudomonas* bacteriophages KPP10, PaP1 and JG004, *Xanthomonas* phage OP2 [[1](#_ENREF_1)] and *Cronobacter* phage CR9 (accession AFH20896, unpublished). However, neither experimental information nor the size and the genomic location of these genes in the structural region could corroborate the hypothesis that the family 28 ORFs could encode this function. In addition, no candidate RNA polymerase was identified in the four bacteriophages belonging to the FelixO1-like genus. By contrast, candidate DNA polymerases (ORFs of the families 66 and 135) were identified with a much higher degree of confidence, on the basis of sequence similarity to many DNA polymerases from other bacteriophages and the presence of a conserved DNA_pol_A domain. Candidates for DNA ligase (families 109 and 194) and RNA ligase (families 60 and 129) functions were also identified. For both families 60 and 129, HHPred analysis revealed that the first half of the query displays similarity to sequences for tRNA-synthetases or tRNA-binding proteins, whereas the second half was similar to RNA-ligase sequences. The proteins of families 65 and 134 were predicted to be primases/helicases, consistent with their location within the DNA replication/metabolism cluster. Candidates for nucleotide metabolism-related enzymes, including a nicotinamide phosphoribosyl transferase and a phosphoribosyl pyrophosphate synthetase (families 101 and 102 respectively) were identified only in PAK_P1, PAK_P2 and PAK_P4. Candidates for dCMP deaminases (family 26) and thymidylate synthases (family 20) involved in dTTP synthesis were found in each of the six genomes which also have candidates for putative alpha and beta subunits of a ribonucleotide reductase enzyme. Interestingly, in PAK_P1, PAK_P2 and PAK_P4, the ORF encoding the beta subunit was located immediately upstream from the ORF encoding the alpha subunit, whereas in PAK_P3, PAK_P5 and CHA_P1, this order was inverted and a gene was inserted between these two ORFs. The inserted gene codes for a 121 (in CHA_P1 and PAK_P3 genomes) or 140 (in PAK_P5) -amino acid long hypothetical protein which is conserved only in the KPP10-like bacteriophages and has no similarity to any other gene or protein present in the databases.

**Annotation of structural proteins**

Following proteomic analysis of the PAK_P3 virion, PAK_P3 gp6 was identified as the major capsid protein, consistent with the presence of a Phage_cap_E conserved domain that was found in all genes of the core family 6. PAK_P3 gp5 displayed a conserved decorating protein domain identified by homology searches despite e-values above the threshold. This head-related function would be consistent with its genomic location immediately upstream from the gene encoding the capsid protein. PAK_P3 gp9 and its homologs constituted core family 8 and displayed similarities to predicted phage head-tail adaptors annotated in bacteria and in SSP1 bacteriophage genomes (probability score: 97.3%). PAK_P3 gp11, a member of core family 9, was identified as a candidate for the tail sheath protein gene. A mutation in this gene was found when the PAK_P3 bacteriophage was adapted to the CHA strain of *P. aeruginosa* [[2](#_ENREF_2)], suggesting that this protein plays a role in host adaptation. The PAK_P3 gp20 (family 31), gp25 (core family 16) and 27 (family 36) proteins also identified by mass spectrometry displayed structural similarity to tail proteins and tail fibers of various bacteriophages. Proteins from core families 11 (including PAK_P3 gp14, identified as component the virion-associated protein by mass spectrometry) and 17 were also found to be related to a tail function. HHPred analysis of PAK_P3 gp22, from family 35, predicted a structural homology to a protein annotated as a putative tail lysozyme. This prediction was supported by the location in the genome of the corresponding gene in the vicinity of the tail and baseplate-related ORFs and gp22 could have a role in the DNA injection function. Surprisingly, this protein had no homolog in PAK_P5, the closest relative to PAK_P3.

A candidate for the tape-measure protein in the genomes of PAK and CHA_P1 group of bacteriophages (families 54 and 119 respectively) was identified with a mean size of 788 aa for PAK_P1, PAK_P2 and PAK_P4 and 778 aa for PAK_P3, PAK_P5 and CHAP1.

Homology searches identified a baseplate-related function for PAK_P3 gp23 (family 120, present only in KPP10-like bacteriophages) and for PAK_P3 gp21 (core family 14), supported by HHPred structural predictions showing similarity to the baseplate assembly protein of coliphage P2 (probability score 99.9% and e value 1e-26).

The three proteins identified by mass spectrometry and found to be outside the structural region of the genomes (gp72, gp160 and gp166), had homologs in all of the KPP10-like bacteriophages but not in the PAK_P1-like bacteriophages. Gp166 was found to have a structure similar to that of the *Bacillus* phage phi26 head fiber protein (probability score > 96%), which is consistent with the structural nature of this protein. Gp72 was found to be encoded by an ORF located near to the ORFs predicted to encode ribonucleotide reductase subunits, and may therefore have a DNA metabolism-related function. Finally, in PAK_P3, the bands corresponding to gp4 (predicted molecular weight 32 kDa) were located at a molecular weight of about 15 kDa on the gel, suggesting possible proteolytic degradation.

**Annotation of packaging-associated functions**

A candidate ORF for a large terminase subunit was identified in the group of PAK and CHA_P1 bacteriophages. No candidate ORF for a small terminase subunit could be identified in PAK_P1-like bacteriophages on the basis of sequence or structural similarities. This may be due to poor conservation of the primary sequences of these proteins or simply to the absence of such a subunit, as was previously reported for other bacteriophages such as FelixO1 [[3](#_ENREF_3)]. However, in KPP10-like bacteriophages, proteins from family 201 were found to be distantly related to proteins annotated as terminases, on the basis of sequence similarity. These proteins displayed structural similarity to proteins annotated as a DNA packaging enzyme and a terminase in bacteriophages SF6 and T4, respectively, with probabilities >99.5 %, suggesting that they may carry out the function of a small terminase.

ORFs encoding 480-amino acid (PAK_P1-like bacteriophages) and 496-amino acid (KPP10-like bacteriophages) proteins, belonging to core family 2, were located immediately downstream from the predicted ORF coding the terminase large subunit. Initial screening against RefSeq detected similarity only to hypothetical proteins, but iterative searches against UniProtKB and structural predictions revealed a distant relationship to ORFs annotated as encoding portal proteins. Finally, ORFs from core family 3 included PaP1_gp048 (G171_gp110) and JG004 ORF PJG4_061, which were both annotated as methyltransferase genes. Methyltransferases have been described in several bacteriophages with different predicted roles. Mostly they serve in protection against host restriction-modification systems, but alternative roles include that of coliphage P1, for which DNA methylation is thought to regulate cleavage at the *pac* site [[4](#_ENREF_4)].

**Annotation of lysis-associated functions**

Homology searches of core family 18 proteins (Table S5) revealed similarities to proteins annotated with peptidoglycan-binding domains or to structures of lysozyme and muramidase. They therefore probably act as endolysins in cell wall degradation, together with a putative holin which is usually encoded by a gene located close by. However, the diversity of holin sequences [[5](#_ENREF_5)] is such that sequence comparisons cannot guarantee accurate identification. In addition, the alignment of ORFs from core family 24 revealed similarities to numerous predicted cell wall hydrolase SleB proteins, and HHPred analyses identified structural similarities to lytic enzymes (hydrolase_2 conserved domain).

**Identification of genome termini**

We applied a strategy based on sequencing coverage to infer the position of genome termini for the six bacteriophages we sequenced [[6](#_ENREF_6),[7](#_ENREF_7)]. A 1170 bp region in PAK_P1, PAK_P2 and PAK_P4 and a stretch of approximately 750 bp in PAK_P3, PAK_P5 and CHA_P1 were found to be highly covered compared to the rest of the sequence (Table S2). The location of this region in PAK_P1, PAK_P2 and PAK_P4 was consistent with the recent experimental determination for the PaP1 genome, these bacteriophages belonging to the PAK_P1-like clade [[8](#_ENREF_8)].

**References**

1. Inoue Y, Matsuura T, Ohara T, Azegami. K (2006) Sequence analysis of the genome of OP2, a lytic bacteriophage of *Xanthomonas oryzae pv. oryzae*. J Gen Plant Pathol: 104-110.

2. Morello E, Saussereau E, Maura D, Huerre M, Touqui L, et al. (2011) Pulmonary bacteriophage therapy on *Pseudomonas aeruginosa* cystic fibrosis strains: first steps towards treatment and prevention. PLoS One 6: e16963.

3. Whichard JM, Weigt LA, Borris DJ, Li LL, Zhang Q, et al. (2010) Complete genomic sequence of bacteriophage felix o1. Viruses 2: 710-730.

4. Skorupski K, Sauer B, Sternberg N (1994) Faithful cleavage of the P1 packaging site (pac) requires two phage proteins, PacA and PacB, and two *Escherichia coli* proteins, IHF and HU. J Mol Biol 243: 268-282.

5. Young R (2002) Bacteriophage holins: deadly diversity. J Mol Microbiol Biotechnol 4: 21-36.

6. Jiang X, Jiang H, Li C, Wang S, Mi Z, et al. (2011) Sequence characteristics of T4-like bacteriophage IME08 benome termini revealed by high throughput sequencing. Virol J 8: 194.

7. Gill JJ, Berry JD, Russell WK, Lessor L, Escobar-Garcia DA, et al. (2012) The *Caulobacter crescentus* phage phiCbK: genomics of a canonical phage. BMC Genomics 13: 542.

8. Lu S, Le S, Tan Y, Zhu J, Li M, et al. (2013) Genomic and Proteomic Analyses of the Terminally Redundant Genome of the *Pseudomonas aeruginosa* Phage PaP1: Establishment of Genus PaP1-Like Phages. PLoS One 8: e62933.
