# Supplementary material for: The Search for Therapeutic Bacteriophages Uncovers One New Subfamily and Two New Genera of Pseudomonas-Infecting Myoviridae
Source: PLoS One. 2015 Jan 28;10(1):e0117163. doi: 10.1371/journal.pone.0117163 (PMC4309531; doi:10.1371/journal.pone.0117163)
Supplement: S1 Table — (PDF) [file pone.0117163.s002.pdf]

**Table S1**: Sequences and coordinates of the boundaries of highly covered regions of the six bacteriophage genomes reported.

| Highly covered regions | | Start | End | Size |
|---|---|---|---|---|
| PAK_P1 | sequence | AGCGA | CGTGGTT | 1170 |
| | coordinates | 67966 | 69139 | |
| PAK_P2 | sequence | AGCGA | CGTGGTT | 1170 |
| | coordinates | 66530 | 67695 | |
| PAK_P4 | sequence | AGCGA | CGTGGTT | 1180 |
| | coordinates | 67395 | 68569 | |
| CHA_P1 | sequence | AGCCATT | CTTGTT | 756 |
| | coordinates | 62708 | 63463 | |
| PAK_P3 | sequence | AGCCATT | CTTGTT | 746 |
| | coordinates | 62771 | 63516 | |
| PAK_P5 | sequence | AGCCATT | CTTGTT | 760 |
| | coordinates | 62171 | 62930 | |
