# Supplementary material for: The Search for Therapeutic Bacteriophages Uncovers One New Subfamily and Two New Genera of Pseudomonas-Infecting Myoviridae
Source: PLoS One. 2015 Jan 28;10(1):e0117163. doi: 10.1371/journal.pone.0117163 (PMC4309531; doi:10.1371/journal.pone.0117163)
Supplement: S2 Table — (PDF) [file pone.0117163.s003.pdf]

**Table S2: Comparison of the codon usage of bacteriophages PAK_P1 and PAK_P3 with that of their host (PAK)**

| | | % of each codon per AA | % of each codon per AA | % codon usage [Phage] / % codon usage [PAK] |
|---|---|---|---|---|
| | | PAK | PAK_P1 | |
| Phe | ttc | 0.95 | 0.75 | 0.79 |
| Phe | ttt | 0.05 | 0.25 | 5.21 |
| Leu | cta | 0.01 | 0.19 | 16.50 |
| Leu | tta | 0.00 | 0.02 | 9.95 |
| Leu | ttg | 0.07 | 0.13 | 1.86 |
| Leu | ctt | 0.03 | 0.19 | 7.29 |
| Leu | ctc | 0.22 | 0.16 | 0.73 |
| Leu | ctg | 0.67 | 0.30 | 0.45 |
| Ile | atc | 0.91 | 0.55 | 0.61 |
| Ile | att | 0.07 | 0.38 | 5.54 |
| Ile | ata | 0.02 | 0.06 | 2.92 |
| Pro | cca | 0.05 | 0.18 | 3.92 |
| Pro | cct | 0.05 | 0.38 | 7.92 |
| Pro | ccc | 0.30 | 0.14 | 0.48 |
| Pro | ccg | 0.76 | 0.29 | 0.39 |
| Tyr | tac | 0.80 | 0.61 | 0.77 |
| Tyr | tat | 0.20 | 0.39 | 1.89 |
| Gln | caa | 0.15 | 0.49 | 3.30 |
| Gln | cag | 0.85 | 0.51 | 0.60 |
| Asn | aac | 0.86 | 0.65 | 0.76 |
| Asn | aat | 0.14 | 0.35 | 2.43 |
| Lys | aaa | 0.13 | 0.42 | 3.34 |
| Lys | aag | 0.87 | 0.58 | 0.66 |
| Asp | gac | 0.80 | 0.57 | 0.71 |
| Asp | gat | 0.20 | 0.43 | 2.19 |
| Glu | gaa | 0.39 | 0.46 | 1.20 |
| Glu | gag | 0.61 | 0.54 | 0.88 |
| Cys | tgc | 0.90 | 0.63 | 0.70 |
| Cys | tgt | 0.10 | 0.37 | 3.75 |
| Trp | tgg | 1.00 | 1.00 | 1.00 |
| Arg | aga | 0.01 | 0.16 | 29.76 |
| Arg | cgt | 0.10 | 0.24 | 2.29 |
| Arg | cgc | 0.65 | 0.17 | 0.26 |
| Arg | cga | 0.03 | 0.16 | 4.96 |
| Arg | cgg | 0.18 | 0.16 | 0.88 |
| Arg | agg | 0.03 | 0.11 | 4.31 |
| Gly | gga | 0.05 | 0.25 | 5.18 |
| Gly | ggt | 0.10 | 0.28 | 2.85 |
| Gly | ggc | 0.73 | 0.30 | 0.41 |
| Gly | ggg | 0.12 | 0.17 | 1.41 |
| | | | | |
| | | | PAK_P3 | |
| Tyr | tac | 0.80 | 0.72 | 0.91 |
| Tyr | tat | 0.20 | 0.28 | 1.35 |
| Gln | caa | 0.15 | 0.37 | 2.49 |
| Gln | cag | 0.85 | 0.63 | 0.74 |
| Asn | aac | 0.86 | 0.78 | 0.91 |
| Asn | aat | 0.14 | 0.22 | 1.51 |

green, codons for which a tRNA is present in PAK_P1 (light green, pseudo-tRNA).
purple, codons for which a tRNA is present in PAK_P3.
yellow, the most frequently used codons.
pink, highest values per amino acid.
beige, lowest values per amino acid.
