# Supplementary material for: The Search for Therapeutic Bacteriophages Uncovers One New Subfamily and Two New Genera of Pseudomonas-Infecting Myoviridae
Source: PLoS One. 2015 Jan 28;10(1):e0117163. doi: 10.1371/journal.pone.0117163 (PMC4309531; doi:10.1371/journal.pone.0117163)
Supplement: S3 Table — (PDF) [file pone.0117163.s004.pdf]

Table S3 Percentage of ORFs homologous between bacteriophages of the PAK_P1-like and KPP10-like clades

Name *(number of predicted ORFs)*	JG004	PAK_P1	PAK_P2	PAK_P4	PaP1	C2_10-Ab1	PAK_P5	CHA_P1	KPP10	LSL4	PAK_P3
JG004 *(161)*	**100**[a]	**89**	**89**	**88**	**83**	**84**	23	24	22	23	24
PAK_P1 *(181)*	**79**	**100**	**90**	**90**	**83**	**78**	24	24	23	24	24
PAK_P2 *(175)*	**82**	**93**	**100**	**95**	**82**	**77**	25	25	23	24	25
PAK_P4 *(174)*	**82**	**94**	**95**	**100**	**83**	**78**	25	25	24	25	25
PaP1 *(157)*	**85**	**96**	**91**	**92**	**100**	**84**	24	24	24	25	25
C2_10-Ab1 *(157)*	**87**	**90**	**86**	**86**	**84**	**100**	24	24	24	25	25
PAK_P5 *(164)*	23	27	26	27	23	23	**100**	**96**	**83**	**94**	**93**
CHA_P1 *(164)*	23	26	26	26	23	23	**96**	**100**	**84**	**93**	**94**
KPP10 *(146)*	25	28	28	29	26	25	**93**	**95**	**100**	**91**	**95**
LSL4 *(165)*	22	27	25	26	24	24	**93**	**92**	**81**	**100**	**91**
PAK_P3 *(165)*	23	27	26	27	24	24	**93**	**93**	**84**	**91**	**100**

[a]: in bold, percentages corresponding to the two clades
