# Supplementary material for: The Search for Therapeutic Bacteriophages Uncovers One New Subfamily and Two New Genera of Pseudomonas-Infecting Myoviridae
Source: PLoS One. 2015 Jan 28;10(1):e0117163. doi: 10.1371/journal.pone.0117163 (PMC4309531; doi:10.1371/journal.pone.0117163)
Supplement: S4 Table — (PDF) [file pone.0117163.s005.pdf]

Table S4 List of the 404 protein families

Family #											
1	CHA_P10001	KPP10_3	LSL40018	PAK_P500001	PAK_P30001	NC_019918_000580	PJG4_059	G171_gp112	PAKP400001	PAK_P200001	PAK_P100001
2	CHA_P10002	LSL40019	KPP10_4	PAK_P500002	PAK_P30002	PAKP400002	PAK_P200002	NC_019918_000590	G171_gp111	PJG4_060	PAK_P100002
3	CHA_P10003	PAK_P500003	PAK_P30003	LSL40020	KPP10_5	G171_gp110	PAK_P200003	PAKP400003	NC_019918_000600	PJG4_061	PAK_P100003
4	CHA_P10004	PAK_P500004	PAK_P30004	LSL40021	KPP10_6	G171_gp109	PAK_P200004	PAK_P100004	NC_019918_000610	PJG4_062	PAKP400004
5	CHA_P10005	PAK_P500005	PAK_P30005	LSL40022	KPP10_7	PAKP400005	PAK_P200005	NC_019918_000620	PJG4_063	G171_gp108	PAK_P100005
6	CHA_P10006	KPP10_8	LSL40023	PAK_P30006	PAK_P500006	NC_019918_000630	PJG4_064	G171_gp107	PAKP400006	PAK_P200006	PAK_P100006
7	CHA_P10007	PAK_P500007	LSL40024	KPP10_9	PAK_P30007	NC_019918_000640	PJG4_065	G171_gp106	PAKP400007	PAK_P200007	PAK_P100007
8	CHA_P10009	PAK_P500009	PAK_P30009	LSL40026	KPP10_11	NC_019918_000660	G171_gp104	PAKP400009	PAK_P200009	PJG4_067	PAK_P100009
9	CHA_P10011	PAK_P500011	LSL40028	KPP10_13	PAK_P30011	PAK_P100011	NC_019918_000680	PAKP400011	PAK_P200011	G171_gp102	PJG4_069
10	CHA_P10012	LSL40029	KPP10_14	PAK_P30012	PAK_P500012	NC_019918_000690	PJG4_071	G171_gp101	PAKP400013	PAK_P200012	PAK_P100012
11	CHA_P10014	KPP10_16	LSL40031	PAK_P500014	PAK_P30014	PAKP400015	G171_gp099	PAK_P200014	NC_019918_000710	PJG4_073	PAK_P100014
12	CHA_P10018	PAK_P500018	PAK_P30018	KPP10_20	LSL40035	G171_gp096	PAKP400019	PAK_P200018	PJG4_077	NC_019918_000740	PAK_P100018
13	CHA_P10019	PAK_P500019	PAK_P30019	LSL40036	KPP10_21	PAK_P100019	PAK_P200019	NC_019918_000760	G171_gp095	PAKP400020	PJG4_078
14	CHA_P10021	PAK_P500021	KPP10_23	PAK_P30021	LSL40038	PAK_P100021	G171_gp093	PAKP400022	PAK_P200021	NC_019918_000780	PJG4_080
15	CHA_P10024	PAK_P500025	KPP10_26	PAK_P30024	LSL40041	PAK_P100024	PAKP400025	PAK_P200024	PJG4_083	G171_gp090	NC_019918_000810
16	CHA_P10025	LSL40042	KPP10_27	PAK_P30025	PAK_P500026	NC_019918_000820	PAK_P100025	G171_gp089	PAKP400026	PAK_P200025	PJG4_084
17	CHA_P10026	PAK_P30026	LSL40043	KPP10_28	PAK_P500027	PAKP400027	PAK_P200026	G171_gp088	PAK_P100026	NC_019918_000830	PJG4_085
18	CHA_P10028	PAK_P500029	PAK_P30028	KPP10_30	LSL40045	G171_gp086	PAKP400029	PAK_P200028	PAK_P100028	NC_019918_000850	PJG4_087
20	CHA_P10066	PAK_P30065	PAK_P500066	KPP10_64	LSL40082	PAK_P100076	PAKP400075	PAK_P200074	PJG4_130	NC_019918_001260	G171_gp048
21	CHA_P10092	PAK_P500092	KPP10_89	PAK_P30092	LSL40109	PAK_P200109	PJG4_161	PAK_P100112	NC_019918_001550	G171_gp017	PAKP400108
22	CHA_P10098	PAK_P500098	PAK_P30099	LSL40115	KPP10_94	NC_019918_001580	G171_gp014	PAK_P100115	PJG4_165	PAK_P200111	PAKP400110
23	CHA_P10103	PAK_P500103	PAK_P30104	LSL40121	KPP10_98	NC_019918_001610	PJG4_167	PAKP400114	G171_gp011	PAK_P100118	PAK_P200115
24	CHA_P10152c	PAK_P500153c	PAK_P30152c	LSL40004c	KPP10_135	G171_gp129	PAK_P100159c	PAKP400153c	PAK_P200154c	NC_019918_000270	PJG4_029
25	CHA_P10155c	KPP10_138	PAK_P500156c	PAK_P30155c	LSL40007c	NC_019918_000340	PJG4_035	G171_gp123	PAKP400159c	PAK_P200160c	PAK_P100166c
26	CHA_P10156c	PAK_P500157c	PAK_P30156c	LSL40008c	KPP10_139	PAKP400156c	PAK_P200157c	PAK_P100163c	PJG4_032	G171_gp126	NC_019918_000310
27	CHA_P10158c	LSL40010c	KPP10_141	PAK_P30158c	PAK_P500159c	PJG4_037	G171_gp122	PAK_P200162c	PAKP400161c	NC_019918_000350	PAK_P100167c
28	CHA_P10008	PAK_P500008	PAK_P30008	LSL40025	KPP10_10	PAKP400008	PAK_P200008	PAK_P100008	PJG4_066	NC_019918_000650	
29	CHA_P10013	PAK_P500013	PAK_P30013	LSL40030	KPP10_15	PAK_P100013	PAKP400014	PAK_P200013	NC_019918_000700	PJG4_072	
30	CHA_P10015	PAK_P30015	LSL40032	KPP10_17	PAK_P100015	G171_gp098	PAKP400016	PAK_P200015	NC_019918_000720	PJG4_074	
31	CHA_P10020	PAK_P500020	PAK_P30020	LSL40037	KPP10_22	NC_019918_000770	PAK_P200020	PAK_P100020	PJG4_079	PAKP400021	
32	CHA_P10032	PAK_P30032	LSL40049	PAK_P500033	PAK_P100032	NC_019918_000880	PJG4_091	G171_gp083	PAKP400033	PAK_P200032	
33	CHA_P10061	LSL40077	PAK_P500061	PAK_P30060	PJG4_118	G171_gp060	PAKP400062	PAK_P100063	NC_019918_001170	PAK_P200062	
34	CHA_P10082	LSL40099	PAK_P500082	KPP10_81	PAK_P30081	G171_gp031	PAKP400092	PAK_P200092	PAK_P100095	NC_019918_001440	
35	CHA_P10022	LSL40039	KPP10_24	PAK_P30022	PAK_P100022	PJG4_081	G171_gp092	PAKP400023	PAK_P200022		
36	CHA_P10027	LSL40044	PAK_P500028	PAK_P30027	G171_gp087	PAKP400028	PAK_P100027	PAK_P200027	NC_019918_000840		
37	CHA_P10105	G171_gp006	PAK_P30106	PAK_P500106	LSL40125	PAK_P100124	PJG4_170	PAKP400118	PAK_P200119		
38	CHA_P10117	LSL40137	PAK_P500116	KPP10_107	PAK_P30118	CHA_P10118	LSL40138	PAK_P500117	PAK_P30119		
39	PJG4_090	PAKP400032	PAK_P200031	PAK_P100031	PJG4_150	G171_gp027	PAKP400096	PAK_P200096	PAK_P100099		
40	PJG4_108	PAK_P200051	NC_019918_001050	PAKP400051	PAK_P100052	PJG4_109	G171_gp070	PAKP400052	PAK_P100053		
41	PJG4_122	PAKP400066	G171_gp056	NC_019918_001200	PAK_P100067	PAK_P200066	PAK_P100068	PAKP400067	PAK_P200067		
42	PJG4_149	PAKP400095	PAK_P200095	NC_019918_001460	PAK_P100098	KPP10_82	LSL40100	PAK_P500083	PAK_P30083		
43	CHA_P10033	PAK_P30033	LSL40050	KPP10_33	PAK_P500034	PAKP400034	PAK_P200033	PAK_P100033			
44	CHA_P10100	LSL40117	KPP10_96	PAK_P500100	PAK_P30101	PAKP400113	PAK_P200114				
45	CHA_P10135	PAK_P500134	KPP10_120	PAK_P30135	PAKP400079	PAK_P200078	PAK_P100080				
46	PJG4_134	PAKP400080	PAK_P200079	PAK_P100081	PAK_P100171c	G171_gp119	PAKP400165c				
47	PJG4_172	PAKP400119	PAK_P100126	KPP10_100	NC_019918_001680	G171_gp005	PAK_P30108				
48	PJG4_001	PAK_P100130	G171_gp001	PAKP400123	NC_019918_001690	NC_019918_000010	PAK_P200124				
49	CHA_P10016	PAK_P500016	PAK_P30016	LSL40033	KPP10_18	PAK_P100016					
50	CHA_P10101	PAK_P500101	KPP10_97	PAK_P30102	LSL40118	PAK_P100119					
51	CHA_P10107	PAK_P500108	LSL40128	PAKP400120	PAK_P200121	PAK_P100128					
52	CHA_P10134	PAK_P500133	LSL40154	KPP10_119	PAK_P100084	G171_gp041					
53	PJG4_068	G171_gp103	PAK_P200010	PAK_P100010	PAKP400010	NC_019918_000670					
54	PJG4_076	G171_gp097	PAKP400018	PAK_P200017	NC_019918_000730	PAK_P100017					
55	PJG4_082	PAK_P200023	NC_019918_000800	G171_gp091	PAKP400024	PAK_P100023					
56	PJG4_088	G171_gp085	PAKP400030	PAK_P200029	NC_019918_000860	PAK_P100029					
57	PJG4_089	G171_gp084	PAKP400031	PAK_P200030	NC_019918_000870	PAK_P100030					
58	PJG4_092	PAKP400035c	NC_019918_000890	PAK_P200034c	PAK_P100034c	G171_gp082					

59	PJG4_094	PAKP400037c	G171_gp081	PAK_P100036c	NC_019918_000910	PAK_P200036c
60	PJG4_096	PAKP400039c	NC_019918_000920	PAK_P200038c	G171_gp079	PAK_P100038c
61	PJG4_101	PAKP400041	PAK_P200041	NC_019918_000950	G171_gp078	PAK_P100041
62	PJG4_104	G171_gp075	PAK_P200045	NC_019918_001000	PAKP400045	PAK_P100045
63	PJG4_105	PAKP400046	G171_gp074	PAK_P200046	PAK_P100046	NC_019918_001010
64	PJG4_106	PAKP400047	PAK_P100047	G171_gp073	PAK_P200047	NC_019918_001020
65	PJG4_110	G171_gp069	PAKP400053	PAK_P200053	PAK_P100054	NC_019918_001070
66	PJG4_111	G171_gp068	NC_019918_001080	PAKP400054	PAK_P100055	PAK_P200054
67	PJG4_113	G171_gp065	PAKP400057	PAK_P200057	NC_019918_001110	PAK_P100058
68	PJG4_114	G171_gp064	PAKP400058	PAK_P200058	NC_019918_001120	PAK_P100059
69	PJG4_115	G171_gp063	PAK_P200059	PAK_P100060	PAKP400059	NC_019918_001140
70	PJG4_116	G171_gp062	PAK_P200060	PAKP400060	NC_019918_001150	PAK_P100061
71	PJG4_117	G171_gp061	PAKP400061	PAK_P200061	NC_019918_001160	PAK_P100062
72	PJG4_119	G171_gp059	PAKP400063	PAK_P100064	NC_019918_001180	PAK_P200063
73	PJG4_121	G171_gp057	PAKP400065	NC_019918_001190	PAK_P200065	PAK_P100066
74	PJG4_124	G171_gp054	PAKP400068	PAK_P200068	NC_019918_001210	PAK_P100069
75	PJG4_127	PAKP400071	PAK_P200071	PAK_P100072	NC_019918_001230	G171_gp051
76	PJG4_131	G171_gp047	PAKP400076	PAK_P200075	PAK_P100077	NC_019918_001270
77	PJG4_132	PAKP400077	PAK_P200076	G171_gp046	NC_019918_001280	PAK_P100078
78	PJG4_133	PAK_P200077	PAKP400078	PAK_P100079	NC_019918_001290	G171_gp045
79	PJG4_135	PAK_P200080	NC_019918_001310	PAKP400081	PAK_P100082	G171_gp043
80	PJG4_136	PAK_P200081	NC_019918_001320	PAKP400082	G171_gp042	PAK_P100083
81	PJG4_139	G171_gp037	NC_019918_001360	PAKP400086	PAK_P200085	PAK_P100088
82	PJG4_140	PAKP400087	PAK_P200086	PAK_P100089	NC_019918_001370	G171_gp036
83	PJG4_143	PAK_P100092	PAKP400090	NC_019918_001400	PAK_P200089	G171_gp033
84	PJG4_146	PAKP400091	PAK_P200091	G171_gp032	PAK_P100094	NC_019918_001430
85	PJG4_148	G171_gp029	PAKP400094	PAK_P200094	PAK_P100097	NC_019918_001450
86	PJG4_151	PAK_P100100	NC_019918_001470	G171_gp026	PAKP400097	PAK_P200097
87	PJG4_153	NC_019918_001480	PAKP400099	PAK_P200100	G171_gp024	PAK_P100103
88	PJG4_156	PAK_P200104	G171_gp022	NC_019918_001510	PAK_P100107	PAKP400102
89	PJG4_160	PAKP400107	PAK_P200108	G171_gp018	PAK_P100111	NC_019918_001540
90	PJG4_162	PAKP400109	PAK_P200110	G171_gp016	PAK_P100113	NC_019918_001560
91	PJG4_166	PAKP400112	PAK_P200113	G171_gp012	PAK_P100117	NC_019918_001600
92	PJG4_169	NC_019918_001640	G171_gp008	PAKP400117	PAK_P200118	PAK_P100122
93	PJG4_004	G171_gp156	PAKP400125c	PAK_P200126c	NC_019918_000020	PAK_P100132c
94	PJG4_005	PAK_P200127c	G171_gp155	PAKP400126c	NC_019918_000030	PAK_P100133c
95	PJG4_007	G171_gp153	PAKP400128c	PAK_P100135c	PAK_P200129c	NC_019918_000050
96	PJG4_009	G171_gp151	PAK_P100137c	PAK_P200131c	PAKP400130c	NC_019918_000070
97	PJG4_010	G171_gp150	PAK_P200132c	PAKP400131c	NC_019918_000080	PAK_P100138c
98	PJG4_011	PAKP400134c	NC_019918_000110	G171_gp148	PAK_P200135c	PAK_P100140c
99	PJG4_012	PAK_P200136c	G171_gp147	PAKP400135c	NC_019918_000120	PAK_P100141c
100	PJG4_016	PAKP400140c	PAK_P100146c	PAK_P200141c	G171_gp142	NC_019918_000150
101	PJG4_018	PAKP400142c	PAK_P100148c	PAK_P200143c	NC_019918_000170	G171_gp140
102	PJG4_020	NC_019918_000190	PAK_P100150c	PAK_P200145c	G171_gp138	PAKP400144c
103	PJG4_022	G171_gp136	PAK_P100152c	PAK_P200147c	NC_019918_000210	PAKP400146c
104	PJG4_023	NC_019918_000220	PAK_P200148c	PAKP400147c	PAK_P100153c	G171_gp135
105	PJG4_024	PAKP400148c	PAK_P200149c	NC_019918_000230	PAK_P100154c	G171_gp134
106	PJG4_026	PAKP400150c	PAK_P200151c	NC_019918_000240	PAK_P100156c	G171_gp132
107	PJG4_028	PAKP400152c	PAK_P200153c	NC_019918_000260	G171_gp130	PAK_P100158c
108	PJG4_030	PAK_P100161c	PAK_P200155c	NC_019918_000290	G171_gp128	PAKP400154c
109	PJG4_031	G171_gp127	PAKP400155c	NC_019918_000300	PAK_P100162c	PAK_P200156c
110	PJG4_033	G171_gp125	NC_019918_000320	PAKP400157c	PAK_P200158c	PAK_P100164c
111	PJG4_034	G171_gp124	PAK_P200159c	PAK_P100165c	NC_019918_000330	PAKP400158c
112	PJG4_038	PAK_P100168c	NC_019918_000360	G171_gp121	PAKP400162c	PAK_P200163c
113	PJG4_041	PAKP400166c	NC_019918_000390	PAK_P100172c	G171_gp118	PAK_P200167c
114	PJG4_042	PAKP400167c	NC_019918_000400	PAK_P200168c	PAK_P100173c	G171_gp117
115	PJG4_043	PAKP400168c	PAK_P200169c	NC_019918_000410	PAK_P100174c	G171_gp116
116	PJG4_044	G171_gp115	PAK_P200170c	PAK_P100175c	NC_019918_000420	PAKP400169c
117	PJG4_045	G171_gp113	PAKP400171	PAK_P200172	NC_019918_000450	PAK_P100177
118	CHA_P10010	PAK_P500010	PAK_P30010	KPP10_12	LSL40027	
119	CHA_P10017	PAK_P500017	PAK_P30017	LSL40034	KPP10_19	

120	CHA_P10023	PAK_P500024	PAK_P30023	KPP10_25	LSL40040
121	CHA_P10029	PAK_P500030	PAK_P30029	LSL40046	KPP10_31
122	CHA_P10035	PAK_P30034	KPP10_34	PAK_P500035	LSL40051
123	CHA_P10036	PAK_P500036	PAK_P30035	LSL40052	KPP10_35
124	CHA_P10039	PAK_P30038	KPP10_37	LSL40055	PAK_P500039
125	CHA_P10041	PAK_P500041	PAK_P30040	LSL40057	KPP10_39
126	CHA_P10042	PAK_P500042	PAK_P30041	LSL40058	KPP10_40
127	CHA_P10043	PAK_P500043	LSL40059	KPP10_41	PAK_P30042
128	CHA_P10044	PAK_P500044	LSL40060	KPP10_42	PAK_P30043
129	CHA_P10045	PAK_P500045	LSL40061	KPP10_43	PAK_P30044
130	CHA_P10046	PAK_P500046	PAK_P30045	LSL40062	KPP10_44
131	CHA_P10047	PAK_P30046	LSL40063	PAK_P500047	KPP10_45
132	CHA_P10048	PAK_P30047	LSL40064	PAK_P500048	KPP10_46
133	CHA_P10049	PAK_P500049	PAK_P30048	LSL40065	KPP10_47
134	CHA_P10050	PAK_P30049	PAK_P500050	LSL40066	KPP10_48
135	CHA_P10051	PAK_P30050	PAK_P500051	LSL40067	KPP10_49
136	CHA_P10052	PAK_P30051	KPP10_50	PAK_P500052	LSL40068
137	CHA_P10053	PAK_P30052	LSL40069	KPP10_51	PAK_P500053
138	CHA_P10054	PAK_P30053	PAK_P500054	LSL40070	KPP10_52
139	CHA_P10055	PAK_P30054	KPP10_53	PAK_P500055	LSL40071
140	CHA_P10056	PAK_P30055	LSL40072	KPP10_54	PAK_P500056
141	CHA_P10057	PAK_P30056	LSL40073	KPP10_55	PAK_P500057
142	CHA_P10058	PAK_P500058	PAK_P30057	LSL40074	KPP10_56
143	CHA_P10059	PAK_P500059	PAK_P30058	LSL40075	KPP10_57
144	CHA_P10060	PAK_P500060	PAK_P30059	LSL40076	KPP10_58
145	CHA_P10062	PAK_P500062	KPP10_60	LSL40078	PAK_P30061
146	CHA_P10063	PAK_P500063	LSL40079	KPP10_61	PAK_P30062
147	CHA_P10064	PAK_P500064	PAK_P30063	LSL40080	KPP10_62
148	CHA_P10065	LSL40081	PAK_P500065	PAK_P30064	KPP10_63
149	CHA_P10067	PAK_P500067	PAK_P30066	LSL40083	KPP10_65
150	CHA_P10068	PAK_P500068	PAK_P30067	LSL40084	KPP10_66
151	CHA_P10069	PAK_P30068	LSL40085	KPP10_67	PAK_P500069
152	CHA_P10070	PAK_P500070	LSL40086	KPP10_68	PAK_P30069
153	CHA_P10071	LSL40087	PAK_P500071	PAK_P30070	KPP10_69
154	CHA_P10072	LSL40088	PAK_P30071	PAK_P500072	KPP10_70
155	CHA_P10073	LSL40089	PAK_P500073	PAK_P30072	KPP10_71
156	CHA_P10074	LSL40090	KPP10_72	PAK_P30073	PAK_P500074
157	CHA_P10075	LSL40091	PAK_P500075	PAK_P30074	KPP10_73
158	CHA_P10077	LSL40093	PAK_P30076	PAK_P500077	KPP10_75
159	CHA_P10078	LSL40094	PAK_P30077	KPP10_76	PAK_P500078
160	CHA_P10079	LSL40095	KPP10_77	PAK_P500079	PAK_P30078
161	CHA_P10080	PAK_P500080	LSL40096	KPP10_78	PAK_P30079
162	CHA_P10081	PAK_P500081	LSL40097	KPP10_79	PAK_P30080
163	CHA_P10085	LSL40102	PAK_P500085	PAK_P30085	KPP10_83
164	CHA_P10086	LSL40103	PAK_P500086	PAK_P30086	KPP10_84
165	CHA_P10087	PAK_P500087	PAK_P30087	LSL40104	KPP10_85
166	CHA_P10088	PAK_P30088	LSL40105	KPP10_86	PAK_P500088
167	CHA_P10091	PAK_P500091	KPP10_88	PAK_P30091	LSL40108
168	CHA_P10093	LSL40110	PAK_P30093	KPP10_90	PAK_P500093
169	CHA_P10095	LSL40112	KPP10_91	PAK_P30096	PAK_P500095
170	CHA_P10096	PAK_P30097	LSL40113	PAK_P500096	KPP10_92
171	CHA_P10097	LSL40114	PAK_P500097	KPP10_93	PAK_P30098
172	CHA_P10099	LSL40116	PAK_P500099	KPP10_95	PAK_P30100
173	CHA_P10108	KPP10_102	PAK_P30111	PAK_P500109	LSL40129
174	CHA_P10115	KPP10_105	PAK_P500114	LSL40135	PAK_P30116
175	CHA_P10116	LSL40136	PAK_P500115	KPP10_106	PAK_P30117
176	CHA_P10120	PAK_P500119	LSL40140	PAK_P30121	KPP10_109
177	CHA_P10121	PAK_P500120	PAK_P30122	KPP10_110	LSL40141
178	CHA_P10122	PAK_P30123	PAK_P500121	LSL40142	KPP10_111
179	CHA_P10124	LSL40144	PAK_P500123	PAK_P30125	KPP10_112
180	CHA_P10125	PAK_P500125	PAK_P30127	LSL40146	KPP10_113

181	CHA_P10126	PAK_P30128	KPP10_114	PAK_P500126	LSL40147
182	CHA_P10129	KPP10_116	PAK_P500129	PAK_P30132	LSL40150
183	CHA_P10139c	PAK_P500140c	PAK_P30139c	KPP10_123	LSL40158c
184	CHA_P10140c	PAK_P500141c	PAK_P30140c	KPP10_124	LSL40159c
185	CHA_P10141c	PAK_P500142c	PAK_P30141c	KPP10_125	LSL40160c
186	CHA_P10142c	PAK_P500143c	LSL40161c	PAK_P30142c	KPP10_126
187	CHA_P10143c	PAK_P500144c	PAK_P30143c	LSL40162c	KPP10_127
188	CHA_P10144c	PAK_P500145c	PAK_P30144c	KPP10_128	LSL40163c
189	CHA_P10145c	PAK_P500146c	KPP10_129	PAK_P30145c	LSL40164c
190	CHA_P10147c	KPP10_130	PAK_P30147c	LSL40166c	PAK_P500148c
191	CHA_P10150c	LSL40002c	PAK_P30150c	KPP10_133	PAK_P500151c
192	CHA_P10151c	KPP10_134	PAK_P30151c	LSL40003c	PAK_P500152c
193	CHA_P10153c	PAK_P500154c	PAK_P30153c	LSL40005c	KPP10_136
194	CHA_P10154c	PAK_P30154c	KPP10_137	LSL40006c	PAK_P500155c
195	CHA_P10157c	PAK_P500158c	KPP10_140	LSL40009c	PAK_P30157c
196	CHA_P10159c	KPP10_142	PAK_P500160c	PAK_P30159c	LSL40011c
197	CHA_P10160c	KPP10_143	PAK_P30160c	LSL40012c	PAK_P500161c
198	CHA_P10162	PAK_P30162	LSL40013	KPP10_144	PAK_P500163
199	CHA_P10163	PAK_P500164	PAK_P30163	LSL40014	KPP10_145
200	CHA_P10164	PAK_P500165	PAK_P30164	LSL40015	KPP10_146
201	CHA_P10165	KPP10_1	PAK_P30165	PAK_P500166	LSL40016
202	CHA_P10166	PAK_P500167	KPP10_2	LSL40017	PAK_P30166
203	PJG4_093	PAKP400036c	PAK_P200035c	PAK_P100035c	NC_019918_000900
204	PJG4_095	PAKP400038c	PAK_P100037c	PAK_P200037c	G171_gp080
205	PJG4_112	PAKP400055	PAK_P100056	G171_gp067	PAK_P200055
206	PJG4_120	G171_gp058	PAKP400064	PAK_P200064	PAK_P100065
207	PJG4_125	PAK_P200069	G171_gp053	PAKP400069	PAK_P100070
208	PJG4_126	G171_gp052	PAK_P200070	PAK_P100071	PAKP400070
209	PJG4_129	PAKP400073	PAK_P100074	PAK_P200073	NC_019918_001250
210	PJG4_138	G171_gp038	PAK_P100087	PAK_P200084	PAKP400085
211	PJG4_141	PAKP400088	PAK_P200087	G171_gp035	PAK_P100090
212	PJG4_154	G171_gp023	PAKP400100	NC_019918_001490	PAK_P100105
213	PJG4_155	PAKP400101	PAK_P200103	PAK_P100106	NC_019918_001500
214	PJG4_158	PAK_P200106	PAKP400105	PAK_P100109	G171_gp020
215	PJG4_159	PAKP400106	PAK_P200107	NC_019918_001530	PAK_P100110
216	PJG4_006	PAK_P200128c	PAKP400127c	NC_019918_000040	PAK_P100134c
217	PJG4_008	PAKP400129c	PAK_P200130c	NC_019918_000060	G171_gp152
218	PJG4_015	PAKP400139c	PAK_P200140c	G171_gp143	PAK_P100145c
219	PJG4_021	PAK_P100151c	PAKP400145c	NC_019918_000200	PAK_P200146c
220	PJG4_025	PAKP400149c	PAK_P200150c	PAK_P100155c	G171_gp133
221	PJG4_027	PAKP400151c	PAK_P200152c	G171_gp131	PAK_P100157c
222	NC_019918_000130	PAKP400136c	PAK_P200137c	G171_gp146	PAK_P100142c
223	NC_019918_000440	G171_gp114	PAKP400170	PAK_P200171	PAK_P100176
224	NC_019918_001100	G171_gp066	PAKP400056	PAK_P200056	PAK_P100057
225	NC_019918_001340	PAKP400084	PAK_P200083	PAK_P100086	G171_gp039
226	NC_019918_001590	PAKP400111	PAK_P200112	G171_gp013	PAK_P100116
227	NC_019918_001630	G171_gp009	PAK_P100120	PAKP400116	PAK_P200117
228	PAK_P100096	G171_gp030	PAK_P200093	PAKP400093	PAK_P30082
229	CHA_P10030	PAK_P500031	PAK_P30030	LSL40047	
230	CHA_P10031	PAK_P500032	PAK_P30031	LSL40048	
231	CHA_P10037	PAK_P30036	PAK_P500037	LSL40053	
232	CHA_P10038	PAK_P30037	LSL40054	PAK_P500038	
233	CHA_P10084	PAK_P500084	PAK_P30084	LSL40101	
234	CHA_P10089	PAK_P500089	PAK_P30089	LSL40106	
235	CHA_P10109	PAK_P500110	LSL40130	KPP10_103	
236	CHA_P10114	PAK_P500113	LSL40134	PAK_P30115	
237	CHA_P10119	LSL40139	PAK_P500118	PAK_P30120	
238	CHA_P10123	LSL40143	PAK_P500122	PAK_P30124	
239	CHA_P10127	PAK_P500127	LSL40148	PAK_P30130	
240	CHA_P10128	PAK_P500128	PAK_P30131	LSL40149	
241	CHA_P10133	PAK_P500132	LSL40153	KPP10_118	

242	CHA_P10137	PAK_P500138	PAK_P30137	LSL40156
243	CHA_P10146c	PAK_P30146c	LSL40165c	PAK_P500147c
244	CHA_P10148c	PAK_P30148c	KPP10_131	PAK_P500149c
245	PJG4_102	PAKP400042	PAK_P200042	PAK_P100042
246	PJG4_128	PAKP400072	PAK_P200072	PAK_P100073
247	PJG4_137	PAKP400083	PAK_P200082	NC_019918_001330
248	PJG4_144	PAK_P200090	NC_019918_001410	PAK_P100093
249	PJG4_152	PAKP400098	PAK_P200098	PAK_P100101
250	PJG4_163	NC_019918_001570	PAK_P100114	G171_gp015
251	PJG4_003	PAK_P200125	G171_gp157	PAKP400124
252	PJG4_017	PAK_P100147c	PAKP400141c	PAK_P200142c
253	LSL40145	PAK_P500124	PAK_P30126	PAK_P100121
254	NC_019918_000090	PAK_P200133c	PAKP400132c	G171_gp149
255	NC_019918_000100	PAK_P200134c	PAK_P100139c	PAKP400133c
256	NC_019918_000180	PAK_P100149c	PAKP400143c	PAK_P200144c
257	NC_019918_000980	PAKP400043	PAK_P200043	PAK_P100043
258	NC_019918_000990	PAKP400044	PAK_P200044	PAK_P100044
259	PAK_P100051	G171_gp071	PAK_P200050	PAKP400050
260	PAK_P100129c	G171_gp002	PAKP400122c	PAK_P200123c
261	PAK_P100170c	G171_gp120	PAK_P200165c	PAKP400164c
262	CHA_P10076	PAK_P30075	PAK_P500076	
263	CHA_P10094	PAK_P500094	PAK_P30095	
264	CHA_P10102	PAK_P500102	PAK_P30103	
265	CHA_P10104	PAK_P30105	KPP10_99	
266	CHA_P10106	PAK_P500107	LSL40126	
267	CHA_P10111c	CHA_P10113c	PAK_P500112c	
268	CHA_P10130	PAK_P500130	PAK_P30133	
269	CHA_P10132	LSL40152	PAK_P500131	
270	CHA_P10136	PAK_P30136	KPP10_121	
271	CHA_P10138c	PAK_P500139c	PAK_P30138c	
272	CHA_P10149c	PAK_P500150c	PAK_P30149c	
273	CHA_P10161	PAK_P30161	PAK_P500162	
274	PJG4_075	PAK_P200016	PAKP400017	
275	PJG4_142	NC_019918_001390	G171_gp034	
276	PJG4_168	G171_gp010	NC_019918_001620	
277	PJG4_171	NC_019918_001670	PAK_P100125	
278	PJG4_036	PAKP400160c	PAK_P200161c	
279	PJG4_040	NC_019918_000380	PAK_P200166c	
280	LSL40127	PAK_P100127	G171_gp004	
281	NC_019918_001650	G171_gp007	PAK_P100123	
282	PAK_P100048	PAKP400048	PAK_P200048	
283	PAK_P100049	PAKP400048.1	PAK_P200048.1	
284	PAK_P100050	PAK_P200049	PAKP400049	
285	PAK_P100091	PAKP400089	PAK_P200088	
286	PAK_P100169c	PAKP400163c	PAK_P200164c	
287	CHA_P10090	KPP10_87		
288	CHA_P10110c	CHA_P10112c		
289	CHA_P10131	LSL40151		
290	PJG4_070	PAKP400012		
291	PJG4_086	KPP10_29		
292	PJG4_103	G171_gp076		
293	PJG4_107	NC_019918_001030		
294	PJG4_123	G171_gp055		
295	PJG4_145	NC_019918_001420		
296	PJG4_014	NC_019918_000140		
297	PJG4_039	NC_019918_000370		
298	PJG4_058	NC_019918_000570		
299	KPP10_74	LSL40092		
300	KPP10_132	LSL40001c		
301	LSL40123	PAK_P500104		
302	LSL40124	PAK_P500105		

303	LSL40131	PAK_P30112.1
304	LSL40132	PAK_P30113
305	NC_019918_000160	G171_gp141
306	NC_019918_000970	G171_gp077
307	NC_019918_001040	G171_gp072
308	NC_019918_001240	G171_gp050
309	NC_019918_001300	G171_gp044
310	NC_019918_001520	G171_gp021
311	PAK_P100085	G171_gp040
312	PAK_P100102	PAK_P200099
313	PAK_P100108	PAKP400103
314	PAK_P100143c	G171_gp145
315	PAK_P100144c	G171_gp144
316	PAK_P100180c	PAK_P200175c
317	PAK_P100181c	PAKP400173c
318	PAK_P200116	PAKP400115
319	PAK_P200122	PAKP400121
320	PAK_P200138c	PAKP400137c
321	PAK_P200139c	PAKP400138c
322	PAK_P30090	PAK_P500090
323	CHA_P10034	
324	CHA_P10083	
325	PJG4_099	
326	PJG4_100	
327	PJG4_147	
328	PJG4_157	
329	PJG4_164	
330	PJG4_173	
331	PJG4_002	
332	PJG4_013	
333	PJG4_019	
334	KPP10_32	
335	KPP10_36	
336	KPP10_59	
337	KPP10_80	
338	KPP10_101	
339	KPP10_104	
340	KPP10_108	
341	KPP10_115	
342	KPP10_117	
343	KPP10_122	
344	LSL40098	
345	LSL40107	
346	LSL40111	
347	LSL40119	
348	LSL40122	
349	LSL40133	
350	LSL40155	
351	LSL40157c	
352	NC_019918_000250	
353	NC_019918_000280	
354	NC_019918_000430	
355	NC_019918_000750	
356	NC_019918_000790	
357	NC_019918_000930	
358	NC_019918_000940	
359	NC_019918_000960	
360	NC_019918_001060	
361	NC_019918_001090	
362	NC_019918_001130	
363	NC_019918_001220	

364	NC_019918_001350
365	NC_019918_001380
366	NC_019918_001660
367	PAK_P100040
368	PAK_P100075
369	PAK_P100104
370	PAK_P100131
371	PAK_P100136c
372	PAK_P100160c
373	PAK_P100178
374	PAK_P100179c
375	PAK_P200101
376	PAK_P200102
377	PAK_P200105
378	PAK_P200120
379	PAK_P200173
380	PAK_P200174
381	PAK_P30107
382	PAK_P30109
383	PAK_P30110
384	PAK_P30112
385	PAK_P30129
386	PAK_P30134
387	PAKP400074
388	PAKP400104
389	PAKP400172
390	PAK_P500015
391	PAK_P500022
392	PAK_P500023
393	PAK_P500111c
394	G171_gp105
395	G171_gp100
396	G171_gp094
397	G171_gp049
398	G171_gp028
399	G171_gp025
400	G171_gp019
401	G171_gp003
402	G171_gp154
403	G171_gp139
404	G171_gp137
