# Supplementary material for: The Search for Therapeutic Bacteriophages Uncovers One New Subfamily and Two New Genera of Pseudomonas-Infecting Myoviridae
Source: PLoS One. 2015 Jan 28;10(1):e0117163. doi: 10.1371/journal.pone.0117163 (PMC4309531; doi:10.1371/journal.pone.0117163)
Supplement: S5 Table — Question marks indicate that only one analysis provided support for the indicated putative function, while absence of question marks indicate that at least two analysis were concordant. (PDF) [file pone.0117163.s006.pdf]

Table S5. Families of core ORFs and families with putative functions

	**Gene identifier**											**Putative function**
	**PAK_P1-like**						**KPP10-like**					
Family Number	PAK_P1	PAK_P2	PAK_P4	JG004	PaP1	C2-10_Ab1	**PAK_P3**	**PAK_P5**	**KPP10**	**CHA_P1**	**LSL4**	
1	*PAK_P100001*[a]	*PAK_P200001*	*PAKP400001*	*PJG4_059*	*G171_gp112*	*YP_007236868.1*	**PAK_P30001**[b]	**PAK_P500001**	**KPP10_3**	**CHA_P10001**	**LSL40018**	Large terminase subunit
2	*PAK_P100002*	*PAK_P200002*	*PAKP400002*	*PJG4_060*	*G171_gp111*	*YP_007236869.1*	**PAK_P30002**[c]	**PAK_P500002**	**KPP10_4**	**CHA_P10002**	**LSL40019**	Portal protein
3	*PAK_P100003*	*PAK_P200003*	*PAKP400003*	*PJG4_061*	*G171_gp110*	*YP_007236870.1*	**PAK_P30003**	**PAK_P500003**	**KPP10_5**	**CHA_P10003**	**LSL40020**	DNA methyltransferase
4	*PAK_P100004*	*PAK_P200004*	*PAKP400004*	*PJG4_062*	*G171_gp109*	*YP_007236871.1*	**PAK_P30004**[c]	**PAK_P500004**	**KPP10_6**	**CHA_P10004**	**LSL40021**	
5	*PAK_P100005*	*PAK_P200005*	*PAKP400005*	*PJG4_063*	*G171_gp108*	*YP_007236872.1*	**PAK_P30005**[c]	**PAK_P500005**	**KPP10_7**	**CHA_P10005**	**LSL40022**	Decorating protein?
6	*PAK_P100006*	*PAK_P200006*	*PAKP400006*	*PJG4_064*	*G171_gp107*	*YP_007236873.1*	**PAK_P30006**[c]	**PAK_P500006**	**KPP10_8**	**CHA_P10006**	**LSL40023**	Major capsid protein
7	*PAK_P100007*	*PAK_P200007*	*PAKP400007*	*PJG4_065*	*G171_gp106*	*YP_007236874.1*	**PAK_P30007**[c]	**PAK_P500007**	**KPP10_9**	**CHA_P10007**	**LSL40024**	
8	*PAK_P100009*	*PAK_P200009*	*PAKP400009*	*PJG4_067*	*G171_gp104*	*YP_007236876.1*	**PAK_P30009**[c]	**PAK_P500009**	**KPP10_11**	**CHA_P10009**	**LSL40026**	Head-tail adaptor
9	*PAK_P100011*	*PAK_P200011*	*PAKP400011*	*PJG4_069*	*G171_gp102*	*YP_007236878.1*	**PAK_P30011**[c]	**PAK_P500011**	**KPP10_13**	**CHA_P10011**	**LSL40028**	Tail sheath protein
10	*PAK_P100012*	*PAK_P200012*	*PAKP400013*	*PJG4_071*	*G171_gp101*	*YP_007236879.1*	**PAK_P30012**[c]	**PAK_P500012**	**KPP10_14**	**CHA_P10012**	**LSL40029**	Structural protein
11	*PAK_P100014*	*PAK_P200014*	*PAKP400015*	*PJG4_073*	*G171_gp099*	*YP_007236881.1*	**PAK_P30014**[c]	**PAK_P500014**	**KPP10_16**	**CHA_P10014**	**LSL40031**	Distal tail protein
12	*PAK_P100018*	*PAK_P200018*	*PAKP400019*	*PJG4_077*	*G171_gp096*	*YP_007236884.1*	**PAK_P30018**[c]	**PAK_P500018**	**KPP10_20**	**CHA_P10018**	**LSL40035**	Tail protein?
13	*PAK_P100019*	*PAK_P200019*	*PAKP400020*	*PJG4_078*	*G171_gp095*	*YP_007236886.1*	**PAK_P30019**[c]	**PAK_P500019**	**KPP10_21**	**CHA_P10019**	**LSL40036**	
14	*PAK_P100021*	*PAK_P200021*	*PAKP400022*	*PJG4_080*	*G171_gp093*	*YP_007236888.1*	**PAK_P30021**[c]	**PAK_P500021**	**KPP10_23**	**CHA_P10021**	**LSL40038**	Baseplate assembly protein
15	*PAK_P100024*	*PAK_P200024*	*PAKP400025*	*PJG4_083*	*G171_gp090*	*YP_007236891.1*	**PAK_P30024**[c]	**PAK_P500025**	**KPP10_26**	**CHA_P10024**	**LSL40041**	
16	*PAK_P100025*	*PAK_P200025*	*PAKP400026*	*PJG4_084*	*G171_gp089*	*YP_007236892.1*	**PAK_P30025**[c]	**PAK_P500026**	**KPP10_27**	**CHA_P10025**	**LSL40042**	Tail fiber?
17	*PAK_P100026*	*PAK_P200026*	*PAKP400027*	*PJG4_085*	*G171_gp088*	*YP_007236893.1*	**PAK_P30026**	**PAK_P500027**	**KPP10_28**	**CHA_P10026**	**LSL40043**	Tail fiber?
18	*PAK_P100028*	*PAK_P200028*	*PAKP400029*	*PJG4_087*	*G171_gp086*	*YP_007236895.1*	**PAK_P30028**	**PAK_P500029**	**KPP10_30**	**CHA_P10028**	**LSL40045**	Endolysin?
20	*PAK_P100076*	*PAK_P200074*	*PAKP400075*	*PJG4_130*	*G171_gp048*	*YP_007236936.1*	**PAK_P30065**	**PAK_P500066**	**KPP10_64**	**CHA_P10066**	**LSL40082**	Thymidylate synthase or thymidylate synthase complementing protein
21	*PAK_P100112*	*PAK_P200109*	*PAKP400108*	*PJG4_161*	*G171_gp017*	*YP_007236965.1*	**PAK_P30092**	**PAK_P500092**	**KPP10_89**	**CHA_P10092**	**LSL40109**	
22	*PAK_P100115*	*PAK_P200111*	*PAKP400110*	*PJG4_165*	*G171_gp014*	*YP_007236968.1*	**PAK_P30099**	**PAK_P500098**	**KPP10_94**	**CHA_P10098**	**LSL40115**	
23	*PAK_P100118*	*PAK_P200115*	*PAKP400114*	*PJG4_167*	*G171_gp011*	*YP_007236971.1*	**PAK_P30104**	**PAK_P500103**	**KPP10_98**	**CHA_P10103**	**LSL40121**	
24	*PAK_P100159*	*PAK_P200154*	*PAKP400153*	*PJG4_029*	*G171_gp129*	*YP_007236848.1*	**PAK_P30152**	**PAK_P500153**	**KPP10_135**	**CHA_P10152**	**LSL40004**	Cell wall hydrolase
25	*PAK_P100166*	*PAK_P200160*	*PAKP400159*	*PJG4_035*	*G171_gp123*	*YP_007236855.1*	**PAK_P30155**	**PAK_P500156**	**KPP10_138**	**CHA_P10155**	**LSL40007**	
26	*PAK_P100163*	*PAK_P200157*	*PAKP400156*	*PJG4_032*	*G171_gp126*	*YP_007236852.1*	**PAK_P30156**	**PAK_P500157**	**KPP10_139**	**CHA_P10156**	**LSL40008**	dCMP deaminase
27	*PAK_P100167*	*PAK_P200162*	*PAKP400161*	*PJG4_037*	*G171_gp122*	*YP_007236856.1*	**PAK_P30158**	**PAK_P500159**	**KPP10_141**	**CHA_P10158**	**LSL40010**	
28	PAK_P100008	PAK_P200008	PAKP400008	PJG4_066		YP_007236875.1	PAK_P30008[c]	PAK_P500008	KPP10_10	CHA_P10008	LSL40025	
29	PAK_P100013	PAK_P200013	PAKP400014	PJG4_072		YP_007236880.1	PAK_P30013[c]	PAK_P500013	KPP10_15	CHA_P10013	LSL40030	Structural protein
31	PAK_P100020	PAK_P200020	PAKP400021	PJG4_079		YP_007236887.1	PAK_P30020[c]	PAK_P500020	KPP10_22	CHA_P10020	LSL40037	Tail protein?

35	PAK_P100022	PAK_P200022	PAKP400023	PJG4_081	G171_gp092		PAK_P30022[c]		KPP10_24	CHA_P10022	LSL40039	Lysozyme
36	PAK_P100027	PAK_P200027	PAKP400028		G171_gp087	YP_007236894.1	PAK_P30027[c]	PAK_P500028		CHA_P10027	LSL40044	Tail fiber
54	PAK_P100017	PAK_P200017	PAKP400018	PJG4_076	G171_gp097	YP_007236883.1						Tape measure protein
60	PAK_P100038	PAK_P200038	PAKP400039	PJG4_096	G171_gp079	YP_007236902.1						RNA ligase?
65	PAK_P100054	PAK_P200053	PAKP400053	PJG4_110	G171_gp069	YP_007236917.1						Primase/helicase
66	PAK_P100055	PAK_P200054	PAKP400054	PJG4_111	G171_gp068	YP_007236918.1						DNA polymerase
77	PAK_P100078	PAK_P200076	PAKP400077	PJG4_132	G171_gp046	YP_007236938.1						Putative ribonucleotide-diphosphate reductase beta subunit
78	PAK_P100079	PAK_P200077	PAKP400078	PJG4_133	G171_gp045	YP_007236939.1						Putative ribonucleotide-diphosphate reductase alpha subunit
101	PAK_P100148	PAK_P200143	PAKP400142	PJG4_018	G171_gp140	YP_007236838.1						Nicotinamide phosphoribosyl transferase
102	PAK_P100150	PAK_P200145	PAKP400144	PJG4_020	G171_gp138	YP_007236840.1						Phosphoribosylpyrophosphate synthetase
109	PAK_P100162	PAK_P200156	PAKP400155	PJG4_031	G171_gp127	YP_007236851.1						DNA ligase
118							PAK_P30010[c]	PAK_P500010	KPP10_12	CHA_P10010	LSL40027	
119							PAK_P30017[c]	PAK_P500017	KPP10_19	CHA_P10017	LSL40034	Tape measure protein
120							PAK_P30023[c]	PAK_P500024	KPP10_25	CHA_P10023	LSL40040	Baseplate protein
129							PAK_P30044	PAK_P500045	KPP10_43	CHA_P10045	LSL40061	RNA ligase?
134							PAK_P30049	PAK_P500050	KPP10_48	CHA_P10050	LSL40066	Primase/helicase
135							PAK_P30050	PAK_P500051	KPP10_49	CHA_P10051	LSL40067	DNA polymerase
155							PAK_P30072[c]	PAK_P500073	KPP10_71	CHA_P10073	LSL40089	
150							PAK_P30067	PAK_P500068	KPP10_66	CHA_P10068	LSL40084	Putative ribonucleotide reductase alpha subunit
152							PAK_P30069	PAK_P500070	KPP10_68	CHA_P10070	LSL40086	Putative ribonucleotide reductase beta subunit
194							PAK_P30154	PAK_P500155	KPP10_137	CHA_P10154	LSL40006	DNA ligase
197							PAK_P30160[c]	PAK_P500161	KPP10_143	CHA_P10160	LSL40012	
201							PAK_P30165	PAK_P500166	KPP10_1	CHA_P10165	LSL40016	Terminase?
202							PAK_P30166[c]	PAK_P500167	KPP10_2	CHA_P10166	LSL40017	Head fiber protein
244							PAK_P30148	PAK_P500149	KPP10_131	CHA_P10148		DNA helicase

[a], italics correspond to the core ORFs of the PAK_P1-like clade

[b], bold correspond to the core ORFs of the KPP10-like clade

[c], proteins identified by mass spectrometry analysis of the PAK_P3 virion
