# Supplementary material for: The Search for Therapeutic Bacteriophages Uncovers One New Subfamily and Two New Genera of Pseudomonas-Infecting Myoviridae
Source: PLoS One. 2015 Jan 28;10(1):e0117163. doi: 10.1371/journal.pone.0117163 (PMC4309531; doi:10.1371/journal.pone.0117163)
Supplement: S1 Fig — A. Representation of the homology (blastn) between the five KPP10-like bacteriophages in which 41-nt repeats were detected (represented as light blue boxes). B. Alignment of the 10 repeats identified in the five genomes, with colors indicating strictly conserved bases, with a WebLogo representation of the consensus (the height of the letters represents their frequency at each position) shown beneath the alignment. (PDF) [file pone.0117163.s007.pdf]

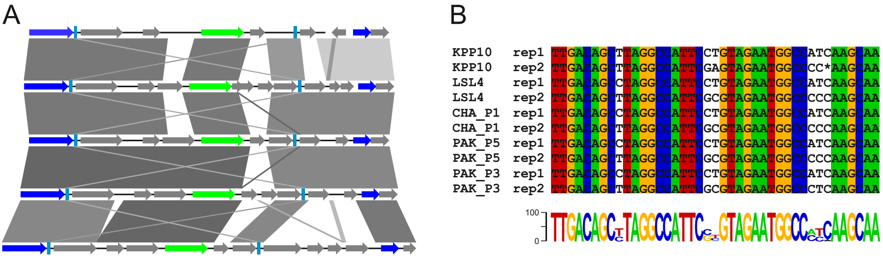

Figure S1: Identification of repeated promoter-like regions of the KPP10-like clade

A. Representation of the homology (Blastn) between the five KPP10-like bacteriophages in which 41-nt repeats were identified (represented as light blue boxes). B. Alignment of the total of 10 repeats identified in the five genomes, with colors indicating strictly conserved bases, with a WebLogo representation of the consensus (the height of the letters represents their frequency at each position) shown underneath.
