# Supplementary material for: The Search for Therapeutic Bacteriophages Uncovers One New Subfamily and Two New Genera of Pseudomonas-Infecting Myoviridae
Source: PLoS One. 2015 Jan 28;10(1):e0117163. doi: 10.1371/journal.pone.0117163 (PMC4309531; doi:10.1371/journal.pone.0117163)
Supplement: S2 Fig — The maximum likelihood tree was built from a protein alignment of the primase sequences, a non-structural protein, common to 19 bacteriophages (the primase was not identified in the PVP-SE1 and GAP31 genomes). Bootstrap support is indicated on the tree. The tree was rooted on the midpoint root. (PDF) [file pone.0117163.s008.pdf]

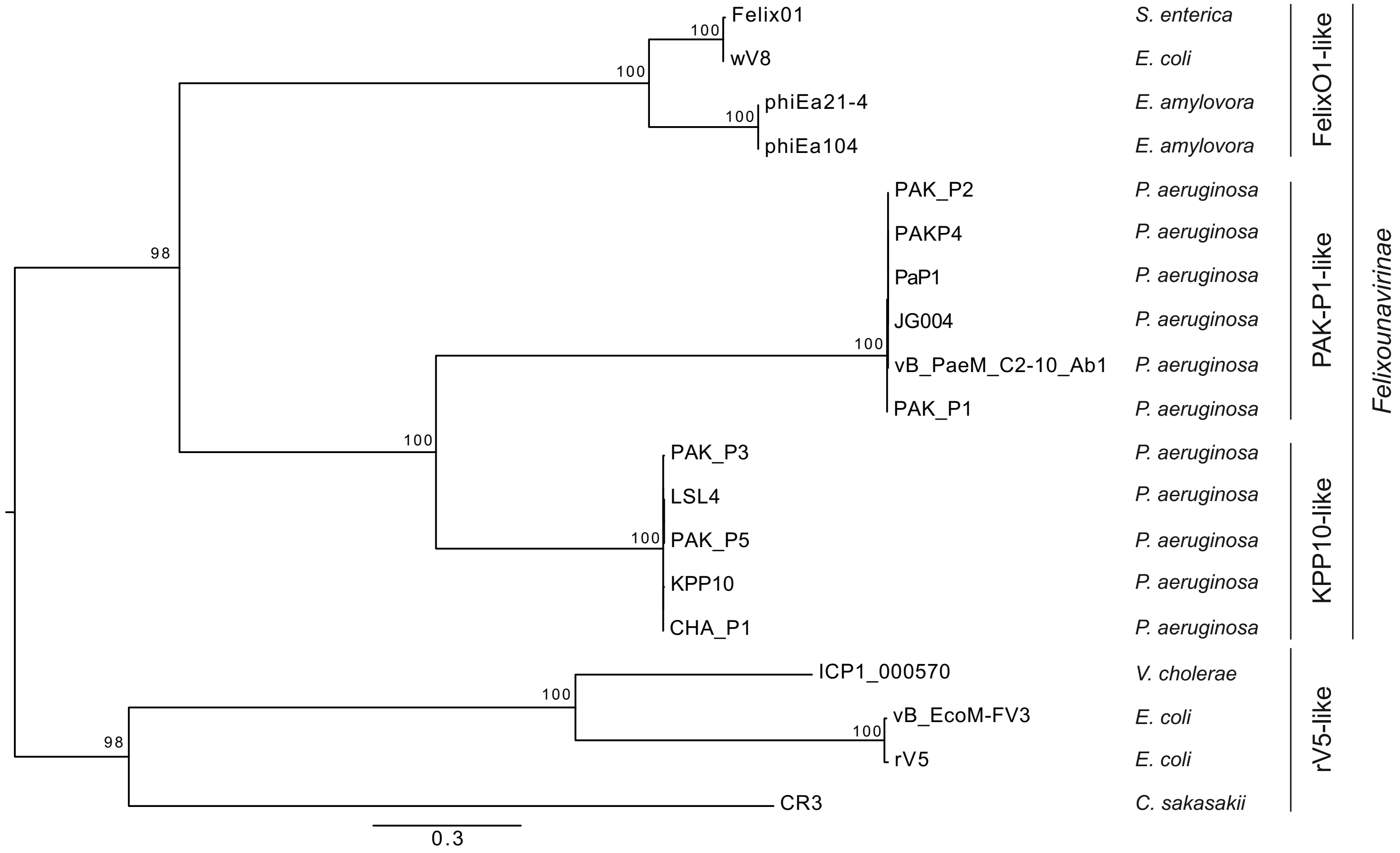

Figure S2: Phylogenetic tree for the primase of PAK_P1-like and KPP10-like bacteriophages and their closest relatives.

The maximum likelihood tree was built from a protein alignment for the primase common to 19 bacteriophages. The primase was not identified in the PVP-SE1 and GAP31 genomes. Bootstrap values are indicated on the tree. The tree was rooted on the midpoint root. The tree is based on the sequence of the primase, a nonstructural protein, and confirms the topology of the tree based on the three concatenated structural proteins.
